# Teaching Direction Control in Horse Riding: A Pre–Post Intervention Proof-of-Concept Study Using Multi-Modal Motor Learning Techniques

**DOI:** 10.3390/ani16203170

**Published:** 2026-10-09

**Authors:** Karin Blignault, Tania Buys, Gopika Ramkilawon, Elize van Vollenhoven

**Affiliations:** 1 Department of Occupational Therapy, Faculty of Health Sciences, School of Health Care Sciences, University of Pretoria, Pretoria 0083, South Africa; apbli@iafrica.com; 2 Private Practice, Cape Town 8001, South Africa; 3 Department of Statistics, Faculty of Natural and Agricultural Sciences, University of Pretoria, Pretoria 0028, South Africa; gopika.ramkilawon@up.ac.za; 4 Department of Companion Animal Clinical Studies, Faculty of Veterinary Science, University of Pretoria, Old Soutpan Road (M35), Onderstepoort, Pretoria 0110, South Africa; elize.vanvollenhoven@up.ac.za

**Keywords:** horse riding, Four-Step Riding Programme, body language, communication, motor learning, multi-modal learning, occupational therapy

## Abstract

Many riders experience persistent communication problems with their horses, particularly in controlling speed and direction, despite extensive training. This proof-of-concept study employed a pre-test–post-test intervention design to examine the effectiveness of the Four-Step Riding Programme (FSRP) in developing clear rider–horse body language for direction control. Multi-modal motor learning techniques (MMLTs) were applied to five rider–horse dyads during four one-hour lessons to teach coordinated movement patterns necessary for directional accuracy. Rider–horse communication was evaluated before and after the intervention using video recordings of 13 riding exercises. Three independent assessors rated the performances on a 5-point Likert scale, and riders provided self-assessments on the same scale. Horse stride parameters, reflecting shoulder control, were visualised with the Alogo™ Move Pro electronic sensor. Significant improvements were observed between pre- and post-intervention scores across all assessors and participants (*p* < 0.05). These preliminary findings suggest that the FSRP holds promise for facilitating the acquisition of rider–horse body language necessary for precise direction control.

## Simple Summary

Controlling a horse’s speed and direction is essential for safe and effective riding. However, many riders struggle to communicate with their horses. When a rider’s body language is unclear, the horse can become confused and anxious, and may react with fight-or-flight behaviours. Modern sport science methods, such as multi-modal motor learning, are not yet part of most horse-riding teaching programs. Instead, instruction often relies on outdated ideas and techniques. This proof-of-concept study tested whether the Four-Step Riding Programme, which incorporates multi-modal motor learning methods, helps riders develop clearer body language to improve speed and directional control. This study aimed to improve rider–horse communication, make riders’ movements smaller and more precise, and observe changes before and after training. Results showed notable improvement in all cases after the intervention. These findings suggest that using this approach can speed up learning for both riders and horses.

## 1. Introduction

Recent events in the sport of dressage, including reports of unethical riding, training, and teaching methods submitted to the Fédération Equestre Internationale (FEI) [1], highlight the urgent need for a systematic approach to rider education that safeguards horse and rider welfare.

Learning to ride is widely recognised as a complex skill that requires extensive practice and time to master [2,3,4]. However, current instructional methods rely largely on historical traditions and anecdotal knowledge [5,6,7], often perpetuating biomechanical misconceptions [8] and outdated practices [3,5,9,10,11]. Despite the prevalence of scientific approaches in other sports, equestrian disciplines remain under-researched [5,12]. Riding instructor accreditation programs also provide limited training in evidence-based motor learning or sports science techniques [11]. As a result, many riders, even after years of lessons, lack fundamental speed and direction control skills [4]. Moreover, riding instruction rarely includes structured methods for learning the coordinated motor skills required to develop clear rider–horse body communication. In contrast, multi-modal motor learning techniques (MMLTs) are well established in coaching, rehabilitation, and other sports disciplines [13,14,15,16].

Over three decades of international teaching experience, the first author observed that riders consistently exhibit similar communication problems with their horses. These include difficulties in stopping, going, bending, maintaining mouth contact, and lateral suppleness. Such issues often stem from a limited understanding of equine and rider biomechanics, as well as ineffective turning techniques, such as pulling the inside rein to turn. Few riders understand that horses need to bend their neck in the direction of movement when riding circles, corners, and turns. Riders also frequently demonstrate mirror movements [17] and arm co-contraction [18], which send conflicting signals to the horse [19]. Conflicting signals can disrupt the horse’s natural balance, leading to incorrect neck flexion, shoulder instability, and confusion. These miscommunications hinder progress, frustrate riders, and increase horse anxiety, which can result in fight-or-flight behaviours [20,21].

Since existing teaching systems have failed to resolve these persistent issues, several researchers have called for a new, evidence-based framework for riding education [5,6,9,10,22,23,24,25,26,27,28]. Given that horse riding presents one of the highest injury rates among sports [29], improving teaching methods is essential for both safety and welfare. This need prompted the development of the Four-Step Riding Programme (FSRP) [19], a structured approach that integrates equine biomechanics, learning theory, and motor learning principles to improve balance, turning, and communication between horse and rider.

Although the broader study addressed both speed and direction control, the present paper focuses on the components of the FSRP that enhance direction control, which depends on lateral and longitudinal equine suppleness. The following sections outline the biomechanical principles of the horse’s head and neck, the fundamentals of equine learning, and the mechanisms of rider–horse body communication, before introducing the structure and motor learning framework of the FSRP.

### 1.1. Biomechanical Functions of the Horse’s Head and Neck

Suppleness is one of the first three elements in the FEI training scale [1,30] and encompasses both longitudinal and lateral components. The increasing use of hyperflexion techniques has prompted extensive research on sagittal-plane flexion and extension of the horse’s head and neck, which reflects longitudinal suppleness [31,32,33]. In contrast, lateral flexion and its influence on the back, limbs, and balance remain underexplored. Direction control is dependent on the horse’s lateral (bend) and longitudinal (flexion throughout the body) suppleness. Suppleness is the innate range of movement, which is genetically determined for each horse [34]. It is dependent on joint mobility, tendon and ligament length, connective tissue and muscle elasticity, is devoid of unnecessary tension, and determines the freedom of movement [34].

Horses typically possess sufficient natural neck flexibility; however, riders often perceive stiffness, which may stem from muscular tension when the horse is cued to perform [34]. The head and neck are integral to the coordination of the entire vertebral column and limbs across different gaits [31,34,35,36,37]. Distinct oscillations of the head and neck correspond to limb movements during a walk, trot, and canter [38].

The head and neck serve as primary mechanisms for maintaining craniocaudal and lateral balance during locomotion [8,31,34]. At liberty, a horse moves its shoulders into the curve and its head and neck to the opposite side for equilibrium [8]. Because the thoracic and lumbar sections of the spine are relatively inflexible [39], lateral bend is limited [34,40], and the horse must take lateral steps to turn, as observed in turns on the forehand and walk pirouettes.

When a rider pulls or opens the inside rein to turn, this natural balance mechanism is disrupted. The horse’s head, neck, and shoulders are drawn inward while the hindquarters swing outward. The resulting lateral step of the inside shoulder, often perceived as “falling in”, occurs because the horse resists rein pressure by bending away from it. Consequently, the horse maintains an outside bend as it turns. To correct this, both horse and rider must learn an alternative turning mechanism in which the horse initiates the turn by bending its own head and neck toward the direction of movement when cued by finger vibrations [8,19]. The horse turns its head and neck with unilateral contraction of its inner neck muscles, as illustrated in Figure 1.

Effective rider instruction must therefore teach coordinated use of the inside leg, body, and arms to guide the horse’s head and neck, while controlling the shoulders.

Consequently, it is important that a riding teaching method synchronises with equine biomechanics and the method by which horses learn most efficiently.

### 1.2. How Horses Learn

Understanding how horses learn and what motivates them is essential for effective, safe, and ethical training [9,42]. Operant conditioning, in which the horse associates its actions and their consequences with specific cues, is the most effective training framework [20]. Learning is reinforced through positive and negative reinforcement [20,43].

Negative reinforcement in animal training involves the removal of aversive stimuli, such as pressure, when a desired response is achieved. In contrast, positive reinforcement involves adding a rewarding stimulus, such as food, to encourage correct behaviour [14,20,44,45]. An acoustic marker, such as a clicker or voice sound (such as a “tsk”), can be used before a treat is offered to clearly communicate that a reward is forthcoming. This can accelerate learning. In riding, negative reinforcement is applied through light, targeted pressure from the rider’s body, which is released immediately when the horse responds correctly. If the response is incorrect, the pressure is gradually increased until the correct reaction occurs. Horses, being sensitive to pressure, quickly learn to respond to minimal cues [20]. Clear, consistent communication minimises confusion and promotes calmness, which optimises learning. Conversely, inconsistent or excessive use of pressure produces anxiety and resistance, undermining training effectiveness.

### 1.3. Rider–Horse Body Language

Rider–horse communication relies on intentional body language based on the application and timely release of pressure [20,46,47,48]. Pressure results from muscle contractions, and its correct use depends on precise coordination and timing. When riders apply pressure cues without appropriate release, or when unintentional muscle contractions occur, signals become inconsistent and ineffective [19].

Each dressage or riding exercise requires the rider to learn a distinct coordinated movement pattern engaging specific muscle groups. Horses can perform these new movements correctly only once the rider achieves sufficient coordination to communicate instructions clearly [19] and avoid conflicting cues [47,48,49,50]. Poor coordination, mistimed releases, or opposing signals may cause confusion.

Repeating exercises under unclear instructions offers little benefit. Because effective rider–horse communication requires the precise coordination of multiple limbs and muscle groups in distinct spatial and temporal patterns [13], instruction must focus on developing independent coordination, controlled muscle activation, and accurate timing [19]. Once these skills are established, riders can guide the horse’s bend and shoulder control during turns, corners, and circles with subtle cues.

The application of MMLT within instruction may enhance this process. MMLT integrates visual, auditory, tactile, kinaesthetic, and proprioceptive feedback to accelerate the acquisition of coordinated motor skills. MMLT is a central component of the FSRP [19].

### 1.4. The Four-Step Riding Programme

The FSRP [19] was developed by the researcher and first author to enhance rider–horse communication by integrating equine biomechanics, equine learning theory, and MMLT into a single teaching framework. The programme is designed to improve both speed and direction control for safe and effective riding through four consecutive one-hour riding sessions. The programme comprises four foundational exercises that underpin all riding movements up to Grand Prix dressage level and form the basis of show jumping.

Figure 2 illustrates how the FSRP exercises develop into the first three elements of the FEI training scale: rhythm and balance, suppleness, and contact [1]. Speed control develops through rhythm and balance, achieved via precise execution of half-halts and alternating between short and long steps. Both of which are dependent on precise executions of stop and go. Direction control depends on developing symmetrical suppleness, which is the ability to bend the neck to both sides with light cues while maintaining straightness through controlled shoulder positioning. Further suppleness is achieved through exercises such as “change of bend at trot” and “leg yield”, which are both derived from the “turn on the forehand” exercise. Consistent, correct execution naturally establishes contact within the flexion frame [19].

The ability to perform the FSRP exercises relies on clear rider–horse body language that the horse can interpret immediately. Mastery of this skill requires independent control of gross and fine motor coordination, visual–motor integration, and motor planning, without mirror movements or co-contraction [19]. MMLT has been shown to provide the optimal pedagogical framework to teach these skills [13,14].

#### 1.4.1. Multi-Modal Learning Techniques Used in the FSRP

The FSRP applies varied practice modes and multi-modal feedback to refine rider coordination and movement accuracy [13]. Once the rider’s motor patterns are optimised, horses can learn corresponding movement patterns effectively, typically within three to six repetitions [52]. The specific motor learning techniques used in the FSRP are: blocked practice, visual demonstration, verbal explanation/cueing and feedback, tactile pressure, kinaesthetic and proprioception feedback, guided movement, a constraints-led approach, external focus, concurrent visual–motor feedback, visualisation, and mental practice to re-inforce the movement pattern. These motor learning techniques are detailed elsewhere [51], but summarized in Figures S1 and S2.

#### 1.4.2. Multi-Modal Teaching Techniques Used in the FSRP

Multi-modal or multi-sensory teaching is highly effective for teaching movement patterns [16]. These methods engage the auditory, visual, tactile, kinaesthetic, and proprioception senses. Furthermore, riders may have unique learning modes. Although verbal instruction is most commonly used in horse riding, it does not reflect physical movement and is among the least effective modes of learning [13]. Too much or too little verbal information about complex movement patterns may be misinterpreted [13].

Teaching effective rider–horse communication, therefore, requires that teachers have a comprehensive understanding of three interconnected concepts. These are: how horses learn, rider–horse body language, and the use of MMLT in teaching.

In summary, the FSRP uses a multi-modal motor learning approach to teach horse riding. However, the effectiveness thereof has not been investigated. The purpose of this pilot study was to determine whether the FSRP could facilitate learning the body language needed for proficient rider–horse communication.

## 2. Materials and Methods

This study was designed as an exploratory proof-of-concept investigation. The sample of five rider–horse dyads reflected the number of eligible participants who could be recruited with comparable presenting problems within the study constraints, with each rider–horse dyad assessed relative to its own pre-intervention baseline. This sample size of five horse–human dyads is consistent with other horse studies using a pre-test–post-test design [53,54,55].

### 2.1. Participants and Infrastructure

A voluntary sampling approach [56] was used to recruit riders who self-identified with communication difficulties with their horses. Invitations were posted in several equestrian social media groups. From 31 qualifying applicants, 6 rider–horse dyads meeting the inclusion criteria were randomly selected to participate in the study (Table 1). Participants for the pilot phase were drawn from the same group.

To ensure ecological validity and accommodate the need for both indoor and outdoor arenas, the study was conducted at a single selected training facility. Due to the prevalence of African Horse Sickness (AHS), only horses in the AHS surveillance zone were permitted to travel to the selected training facility. Limited overnight housing, animal husbandry, and difficulty with daily travel further limited the sample size to five participants. Only one male rider applied to participate, but he was outside the study’s age limit. The sample was thus restricted to female participants.

Participants completed a baseline questionnaire that captured individual variables, including years of riding experience, years of riding lessons, number of riding instructors, rider age, horse age, and years the rider had ridden the current horse (Table 2). Each participant had received instruction from multiple riding teachers throughout their riding careers. These ranged from three to eight instructors. The horses were provided with haylage ad libitum and concentrates three times daily. They were groomed twice daily and housed in 4 × 4 m stables overnight. During the day, the horses were kept in a secure paddock with other horses. Ethical approval was granted by the University of Pretoria, Faculty of Health Sciences Research Ethics Committee and the Animal Ethics Committee. Pseudonyms were used for all rider–horse dyads.

Details on individual horse and rider demographics can be found in Table S1.

### 2.2. Data Collection

This study was conducted at a designated training facility with both indoor and outdoor arenas. The arena surfaces consisted of leather chips, river sand, and clean wood shavings for optimal shock absorption.

To ensure validity, environmental conditions were standardised across all sessions. Horses were transported to the facility by their owners and allowed to acclimate to both arenas before pre-intervention assessments. The two-hour acclimation period was based on the general adaptation period for riders arriving at competition events or new external riding courses. Each horse was stabled on-site for three nights. The FSRP assessments took place in the indoor arena, and the GPS-based assessments were conducted outdoors to improve connectivity.

Thirteen data points were used to assess direction control. Data were collected using three primary tools:

1. Video analysis: Recordings of 13 exercises were evaluated using a 5-point Likert scale.
2. Self-assessment: Riders rated their pre- and post-intervention performance on a 5-point Likert scale.
3. Alogo™ Move Pro analysis: The Alogo™ Move Pro electronic sensor (Alogo Swiss Technology, Renens, Lausanne, Switzerland) was used to visualise horse stride parameters, rhythm, and balance.

Videos were recorded using an iPhone 13 from multiple fixed points around the arena (Figure 3a,b). Most exercises were performed on a marked circle, while the “walking straight with a bend” exercise was completed on a 12 m straight line marked at 3 m intervals along the centre line. The “change of the bend at trot” exercise was recorded from the side of the arena between two interconnected circles (Figure 3b).

The Alogo™ sensor, positioned at the centre of the girth, recorded stride dynamics via a satellite connection to track changes in movement during bend transitions. Changes in stride were depicted using blue dots. Closer dots show short strides, whereas dots farther apart show longer strides. The direction of the dots shows the direction of the limb movements, which indicate shoulder control. The red dot in some of the images indicates speed (meters per minute). Speed was not assessed because this study focused on circles and bend-change exercises. The light green dots generally indicate walk strides, but very short, slow trot strides could be interpreted as walk strides. This visual output from the Alogo™ system illustrates the overall changes in circle shape and bend transitions between the pre- and post-intervention assessments.

Three independent assessors, each an FEI-certified coach, national dressage and equitation judge, and South African Sports Coach Association-certified national coach developer, evaluated all recordings using a standardised 5-point Likert scale. Inter-rater reliability was maintained through the use of a detailed scoring guideline (Figures S3 and S4). Each assessor independently judged the same video footage of each exercise. The participants were anonymised and identified by Greek letters. Each assessor assessed 25 sets of pre-, daily, and final post-intervention video recordings per participant, with 1- to 2-week intervals.

### 2.3. Procedures

Each session began with pre-intervention assessments of 13 variables: change of bend left and right; lateral suppleness on the bend changes left and right; leg yield left and right; rein contact in a round frame at trot; rein contact in a round frame at walk; turn on the forehand right and left; walk to trot contact in a round frame; and walking straight with a bend left and right (See Figure S5 for the 13 variables and Section S1 for the description of the exercises).

Participants then completed four one-hour lessons held on consecutive days, each covering the four FSRP foundation exercises. The first lesson followed a standardised sequence for each rider, while subsequent sessions addressed individual coordination issues identified during assessments. Table 3 describes the FSRP and standardisation processes.

Each rider–horse dyad exhibited unique learning profiles in coordination, learning speed, and communication, which were addressed through tailored combinations of MMLT techniques. To minimise dual-task interference, instructions were provided at the halt, integrating verbal explanations, visual demonstrations, and tactile, kinaesthetic, and proprioceptive techniques. Exercises began with walking to reduce balance disruptions.

All MMLT techniques and feedback were applied across all exercises and participants. Please see Figures S1 and S2 for an explanation of the MMLT techniques and feedback used and their application within the FSRP. Any minor deviations made to assist a particular participant in understanding or performing an exercise were recorded (see Table S2). To ensure consistent application of the MMLT techniques and feedback throughout the study, the researcher was the sole coach.

### 2.4. Method

The intervention focused on teaching specific coordinated motor skills required for accurate timing of pressure-release cues, correct muscular engagement, and elimination of contradictory signals, co-contraction, and mirror movements. Exercises followed a structured progression from simple to complex tasks.

The programme began with a 30 min lecture that explained equine anatomy and biomechanics to establish a theoretical understanding [8,19]. Positive reinforcement using clicker training was also introduced to each horse. The full description of the exercises can be found in Section S1. A simplified description of the various exercises associated with the FSRP is provided in Table 4, together with definitions of the riding terminology.

**Table 4:** Simplified summary of the exercises included in the Four-Step Riding Programme (FSRP) relevant to direction control.

FSRP Exercises **	Main Outcome	Horse’s Actions	Rider’s Actions	Multi-Modal Learning
The turn on the forehand (lateral displacement of the hindquarters around the forelimbs).	Controls shoulders and hindquarters.	Sequential lateral steps with hindlimbs around static forelimbs.	Single leg pressures. Hands connected on wither.	Blocked practice, visual demonstration, verbal explanation/cueing and feedback, tactile pressure, kinaesthetic and proprioception feedback, guided movement, a constraints-led approach, external focus, concurrent visual–motor feedback, visualisation, mental practice to reinforce the movement pattern.
Walking straight with a bend (walking straight with lateral head and neck flexion).	Control shoulders of horse during head and neck bend while the horse is walking in a straight line.	Walks in a straight line while maintaining lateral flexion. Change the bend on a straight line. Turn only when the bend can be maintained while walking straight.	Inside-leg *** pressure and vibration of the inside *** rein. Turn by bringing the outside rein closer to the neck.	
Yielding in a round flexion frame—contact (longitudinal flexion of body).	Horse in a flexed neck and head position.	Horse flexing neck and head (longitudinal flexion).	Finger vibrations through the reins, and release of pressure once the horse responds.	
The leg yield with the horse’s head to the wall for shoulder control (horse taking steps laterally).	The horse steps laterally at a 45° angle to the wall in the arena.	The horse steps laterally with its forelimbs and hind limbs.	Inside-leg *** pressure. Hands interlocking, controlling the shoulders.	
Trotting two 20 m circles with a change of bend in between the two circles for shoulder control and head and neck bend (trotting two 20 m circles with a change of lateral body flexion and direction between the two circles).	Control of the horse’s shoulders during head and neck bend, while changing direction and lateral flexion between the two circles.	Changes in direction and lateral flexion of the neck between the two circles.	Changes to new inside-leg *** pressure and new inside-rein *** vibrations. Turn by bringing the outside rein closer to the neck.	

** The FSRP exercises are associated with direction control in horse riding. In brackets is the scientific explanation. *** Inside refers to the side of the horse facing the centre of the circle or the direction towards which the horse is turning, and outside to the opposite side.

An in-depth explanation of these exercises can be found in Section S1. Refer to Figure 4, Figure 5, Figure 6, Figure 7 and Figure 8 for visual representations of the exercises.

To summarise, all participants were taught the same four basic exercises during the first lesson and practised these exercises during the following three lessons (see Table 4, Figure 4, Figure 5, Figure 6, Figure 7 and Figure 8, and Section S1), using multi-modal motor learning techniques and feedback (Figures S1 and S2) adapted to the individual participant’s needs. Contact in the round flexion frame was taught to all riders during the third lesson and consolidated during the fourth lesson.

The assessors then evaluated video recordings of the riders performing these exercises using 13 predefined variables (see Figure S5).

### 2.5. Statistical Analysis

Statistical analyses were performed in R (version 4.2.1). Since the direction-control variables were scored on a 5-point ordinal Likert scale, non-parametric methods were used throughout. Inter-rater reliability among the three assessors was assessed separately for the pre- and post-intervention scores of each rider–horse dyad using a two-way, average-measures intraclass correlation coefficient (ICC) based on absolute agreement. ICC estimates were interpreted according to the criteria of Koo and Li [57], with values below 0.50, from 0.50 to 0.75, from 0.75 to 0.90, and above 0.90 indicating poor, moderate, good, and excellent reliability, respectively.

The three assessor scores were averaged to obtain a mean assessor score for each of the 13 direction-control variables within each rider–horse dyad. For each rider–horse dyad, the pre- and post-intervention mean assessor scores were then compared across the 13 variables using the paired Wilcoxon signed-rank test. The rider–horse dyads were treated as the unit of replication, and the results were interpreted primarily according to the consistency of the direction of change across the five combinations rather than on the basis of any single *p*-value. For each test, the rank-based effect size, r = Z/√N, was computed and reported alongside the *p*-value, so that the magnitude of the pre- to post-intervention change could be judged independently of statistical significance. Participants additionally completed self-assessments of their direction-control performance before and after the intervention; these paired scores were analysed using the paired Wilcoxon signed-rank test in the same manner. All analyses were conducted at a 5% significance level.

## 3. Results

### 3.1. Inter-Rater Agreement

The ICC assessed inter-rater reliability for pre- and post-intervention scores (Table 5). Except for Gamma and Epsilon’s pre-intervention scores and Digamma’s post-intervention scores, ICC values showed moderate to good reliability. The elevated *p*-value for Digamma resulted from one assessor consistently awarding the maximum score (5) post-intervention, while the other assessors rated more conservatively (mostly 4s).

### 3.2. Pre-Intervention Versus Post-Intervention Scores: Assessors

Table 3 presents the Wilcoxon test results and effect sizes for each participant as rated by the three assessors. All scores improved significantly following the implementation of the FSRP (Figure 8). Large effect sizes across all comparisons confirmed both statistical and practical significance, demonstrating gains in suppleness, bend, and shoulder control.

The magnitude of the descriptive change varied between combinations, with Beta showing the largest increase of 2.44 points and Delta the smallest increase of 1.10 points off a markedly higher pre-intervention baseline of 3.72. As shown in Table 6, the increase was consistent across all 13 measured variables within every rider–horse dyad, and this within-combination improvement was accompanied by large effect sizes throughout. Because each rider–horse dyad comparison was based on 13 measurements that improved in a near-uniform direction, the effect size for that comparison is compressed toward its upper bound and should be read as reflecting the consistency of the change across the measured variables rather than a finely graded magnitude.

The assessor-rated scores, comprising 13 variables per combination, are shown as boxplots to convey their distribution (Figure 9).

### 3.3. Pre-Intervention Versus Post-Intervention Scores: Self-Assessments

According to the participants, the intervention significantly improved their direction control (Table 7, Figure 10). One participant (Gamma) perceived a slight improvement in direction control. The effect size was large for all participants (Table 7).

Four of the five participants showed significant increases in self-assessment scores from pre- to post-intervention. Significant increases were observed for Beta, Delta, Epsilon, and Digamma, whereas the change for Gamma was not significant. Effect sizes were large for all five participants, ranging from r = 0.7628 to r = 0.9258. As with the assessor-rated scores, the large effect sizes should be interpreted in the context of the small number of paired observations and the consistency of the direction of change, rather than as evidence of statistical significance alone.

### 3.4. Stride Parameters

The Alogo™ Pro GPS sensor provided stride-tracking visualisations, with each trot stride represented by a blue dot. The data illustrated the transformation in stride rhythm, symmetry, and bend execution between pre- and post-intervention sessions.

Satellite imagery illustrates the comparative stride patterns before and after intervention. Data for participant Beta were unavailable due to poor satellite connectivity during that session.

### 3.5. Individual Results

#### 3.5.1. Beta’s Results

Beta showed significant improvement in all evaluated aspects of direction control, including contact (flexion), suppleness, bending, and shoulder control. Satellite data from the Alogo™ Move Pro were unavailable due to connectivity issues during Beta’s sessions.

In Figure 11a (pre-intervention), Beta is shown at the trot in an extended, hollow frame with minimal longitudinal or lateral suppleness. The rider’s open right arm indicates a compensatory attempt to steer rather than communicate a bend through the body. In contrast, Figure 11b (post-intervention) shows a clear improvement in flexion, suppleness, and shoulder control. Visible contraction of the horse’s inner neck muscles and a soft, looped inside rein indicate true lateral flexion and self-carriage. The soft, round contact further demonstrates enhanced longitudinal suppleness.

#### 3.5.2. Gamma’s Results

As shown in Figure 12 and Figure 13, Gamma demonstrated significant improvement in all exercises related to contact (flexion), suppleness, bending, and shoulder control, as assessed by the assessors.

Gamma’s pre-intervention self-assessments did not align with assessor scores. After the intervention, the rider recognised that her initial evaluations overestimated performance. When cued to rescore her pre-intervention performance using her improved understanding, her results correlated closely with assessor scores. She remarked, “My mindset on what is adequate in a movement has greatly changed”. This shift reflected a possible Dunning–Kruger effect, supported by a pre-test versus post-test *p*-value of 0.031 and a pre-test versus retest versus post-test *p*-value of 0.0083.

In Figure 12a, Gamma is shown on a left circle with a hollow frame, high head carriage, and limited shoulder control, indicating the absence of true bend. Post-intervention Figure 12b, Gamma demonstrates a rounded flexion frame with improved balance, suppleness, and the ability to bend his neck in the direction of movement.

Gamma’s pre-intervention satellite image (Figure 13a) shows negligible bend change, irregular rhythm, and poor shoulder control during the right-to-left change of bend. Post-intervention (Figure 13b), stride patterns became more even, with improved balance, rhythm, and lateral suppleness. Similar improvements are evident between Figure 13c,d, illustrating enhanced shoulder control and rhythm on the left rein circle.

#### 3.5.3. Delta’s Results

As shown in Figure 14 and Figure 15, Delta demonstrated significant improvement in rein contact, suppleness, bend, and shoulder control.

In the pre-intervention photo (Figure 14a), Delta shows minimal lateral bend despite the rider’s attempt to pull the rein, with no visible contraction of the internal neck musculature. Post-intervention (Figure 14b), contraction of the horse’s brachiocephalicus, splenius, sternocephalicus, and semi-spinalis muscles demonstrated self-initiated bending and engagement in a round flexion frame with light contact.

The pre-intervention satellite image (Figure 15a) reveals a loss of rhythm, balance, and shoulder control during the right-to-left change of bend, with no clear lateral flexion transition. Post-intervention (Figure 15b) images show even stride spacing and a distinct, coordinated change of bend. Similarly, Figure 15c,d illustrate a marked improvement in the shape and consistency of Delta’s left circle, reflecting improved rhythm, balance, and shoulder control.

#### 3.5.4. Epsilon’s Results

Figure 16 and Figure 17 illustrate significant improvement after the intervention. In Figure 16a, Epsilon appears in a hollow head and neck extensor frame with a high head carriage and tight rein, indicating resistance to lateral flexion. The post-intervention photograph (Figure 16b) shows activation of the inside neck muscles and a soft, yielding inside rein, reflecting improved contact, suppleness, and balance.

Pre-intervention satellite data (Figure 17a,c) show rhythm disruptions, loss of balance, and inconsistent bends during the change-of-bend and circle exercises. Post-intervention satellite images (Figure 17b,d) show markedly improved stride spacing and circle symmetry, indicating better shoulder control, rhythm, and balance.

#### 3.5.5. Digamma’s Results

As shown in Figure 18 and Figure 19, Digamma demonstrated significant improvement across all parameters, including rein contact, suppleness, bend, and shoulder control. In Figure 18a, the pre-intervention photograph shows Digamma appearing in a hollow frame with a high head carriage and limited engagement. The rider’s open left arm, tight rein, and absence of muscle definition indicate a lack of bend. Post-intervention (Figure 18b), Digamma shows a rounded flexion frame, active engagement of the neck musculature, and improved shoulder control. The visible positive advanced diagonal hoof placement, where the left hind lands ahead of the diagonal right fore, demonstrates increased hindquarter engagement and forward propulsion. Pre-intervention, Digamma showed negative advanced diagonal hoof placement, indicating disengagement (Figure 18a).

Digamma’s pre-intervention satellite image (Figure 19a) reveals a straight-line diagonal track between circles, indicating no true change of bend. The post-intervention image (Figure 19b) shows an apparent change in bend with the horse slightly side-stepping to ensure the bend change. Although Figure 19c,d show similar circle shapes, post-intervention rhythm and tempo improved noticeably.

## 4. Discussion

This proof-of-concept study investigated the acquisition of rider motor skills needed to develop clear rider–horse communication and achieve effective horse direction control. The FSRP exercises were employed to facilitate this process. The findings demonstrated that the FSRP, incorporating MMLT, may enable riders to develop coordinated motor skills essential for clear communication with their horses. The riders in this study acquired skills that enhanced their ability to teach their horses basic exercises underpinning dressage and show-jumping movements. Within four one-hour sessions held on consecutive days, participants achieved measurable improvements in lateral and longitudinal suppleness, bending, and shoulder control. Agreement among assessors and participants, supported by Alogo™ Move Pro sensor data, suggests that the FSRP may be effective for teaching direction control in horse riding.

Before the intervention, the participants displayed deeply ingrained incorrect motor habits that produced ambiguous rider–horse cues. These issues were reflected in both pre-intervention questionnaires and assessments. All riders pulled back on the reins when cueing their horses to move forward from a halt, relied on opening the reins rather than on small finger vibrations to turn (disrupting the horse’s lateral balance), and did not understand how to initiate bending or lateral movement, which is a basic requirement of horse control. Riders exhibited mirror movements of both legs and ipsilateral arm–leg coordination errors during lateral work, along with arm co-contraction. None of the riders understood that the outside rein governs shoulder control or how to achieve lateral flexion without the horse losing balance and taking a large inward step. Moreover, all participants were unfamiliar with establishing contact in the flexion frame, a prerequisite for harmony, acceptance of the bit, submission, and pliability. None of the participants could execute basic shoulder and hindquarter control exercises, such as turning on the forehand. Across horses, contact resistance and mouth tension were consistently observed.

All of these problems were addressed through the FSRP. None of the participants understood that negative reinforcement refers to the release of pressure following a correct response. This misunderstanding led them to maintain pressure rather than yield, and attribute contact problems to bit design rather than inconsistent signals. Integrating motor learning principles with the correct application of positive and negative reinforcement enabled riders to provide precise feedback to their horses.

Rectification of errors across all 13 evaluated performance variables demonstrated consistent improvement in communication among all participants, regardless of horse breed, rider or horse age, riding experience, or number of previous instructors. These results challenge the common assumption that mastering effective rider–horse communication requires many years of training. Instead, this study suggests that evidence-based MMLT may help teach that foundational riding skills can be acquired rapidly and effectively.

The horses appeared less anxious and learned quickly, suggesting they were less confused by inconsistent rider cues, which were markedly reduced. This supports the FEI’s vision of happy athletes by promoting welfare through improved understanding and communication.

This study has several limitations. The sample size was small, comprising only five rider–horse dyads. Each rider–horse dyad was assessed relative to its own pre-intervention baseline. The findings cannot be generalised beyond this sample. As this was a preliminary study, the satellite data were analysed qualitatively. Given the exploratory nature of the study and the small sample size, the findings are considered preliminary and are not intended to provide definitive evidence of intervention effectiveness. To account for the inherent variability among horses and riders, each horse–rider dyad was assessed relative to its own pre-intervention baseline, thereby ensuring that the same combination of variables was evaluated consistently. Improvements in directional control, through the development of lateral bend and shoulder control, showed that learning could be enhanced in a short period. The assessors and participants agreed that performance improved, and the Alogo™ Move Pro sensor satellite images further corroborated improved shoulder control. Although progressive acclimatisation to the new stable environment may have influenced the results (pre-test vs. post-test) to some extent, it is unlikely to fully explain the improvement as the participants reported these riding difficulties even during instructions at the horses’ usual stables, where they were fully habituated to the environment.

Future studies could build on these findings by addressing these limitations, such as by including male riders, and including objective physiological and behavioural measures to assess equine anxiety and welfare. The inclusion of a control group would have strengthened the interpretation of the results.

Thus, it is recommended that further research with larger groups, using a more robust experimental design and rigorous assessor blinding that includes control groups, be conducted.

## 5. Conclusions

These findings suggest that the FSRP, using MMLT, may facilitate the learning of rider–horse body language needed for achieving direction control through suppleness, bend, and shoulder control.

This study suggests that only four one-hour lessons were needed to improve riders’ coordinated motor patterns and establish the first three elements of the FEI training scales. These findings suggest that foundational rider–horse communication skills can be acquired far more rapidly than traditionally assumed. Aligning rider teaching strategies with the principles of how horses learn, through appropriately timed pressure and release, may reduce confusion, anxiety, and defensive reactions, thereby enhancing equine welfare.

## Figures and Tables

**Figure 1:**
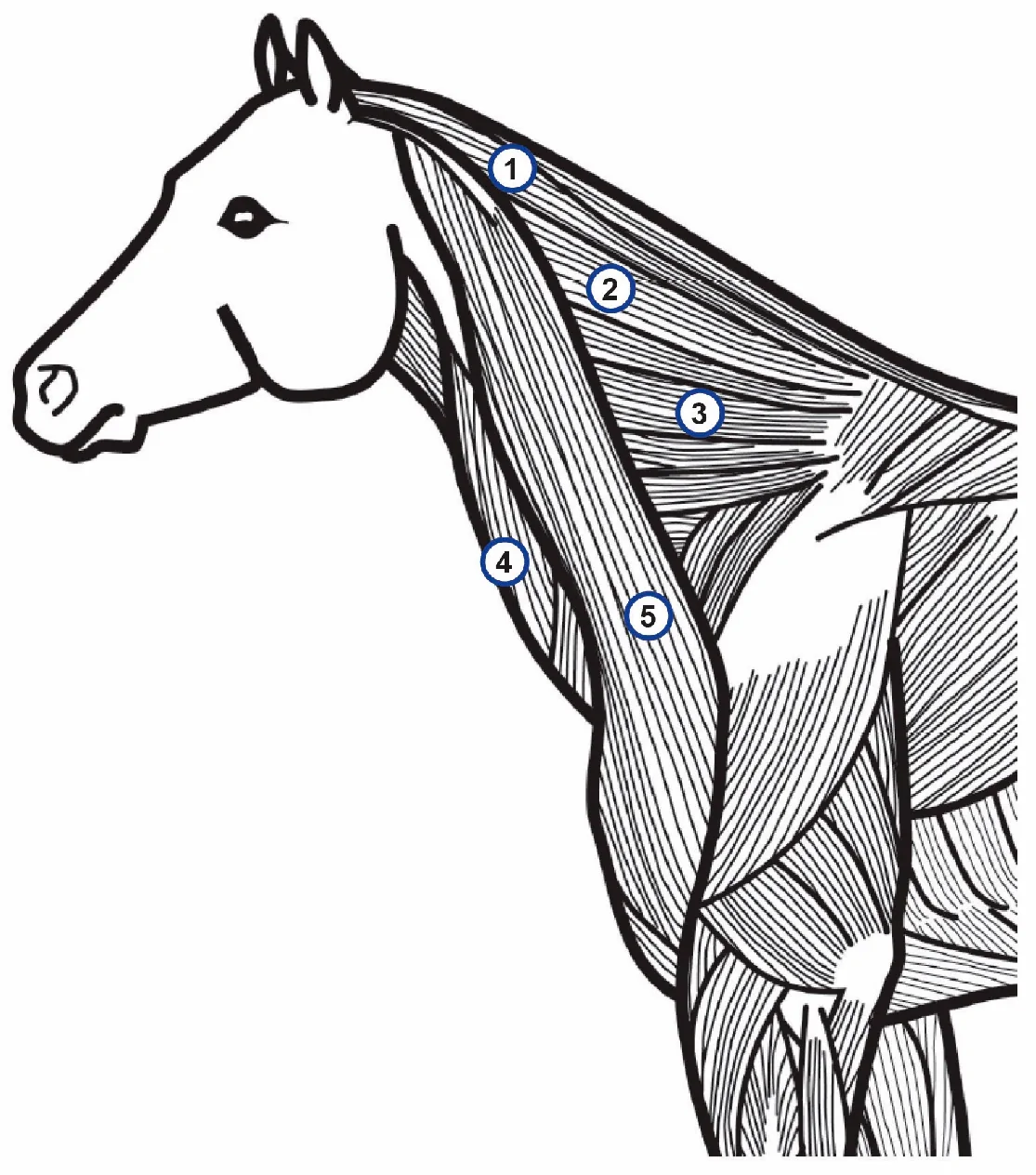
Unilateral neck muscles contracting for lateral flexion (bend) of the horse’s head and neck, including the (1) semispinalis capitis, (2) splenius, (3) serratus ventralis cervicis, (4) sternocephalicus, and (5) brachiocephalicus. Reproduced with permission from K. Blignault, Stretch Exercises for Your Horse: The Path to Perfect Suppleness; published by Trafalgar Square, 2013 [41].

**Figure 2:**
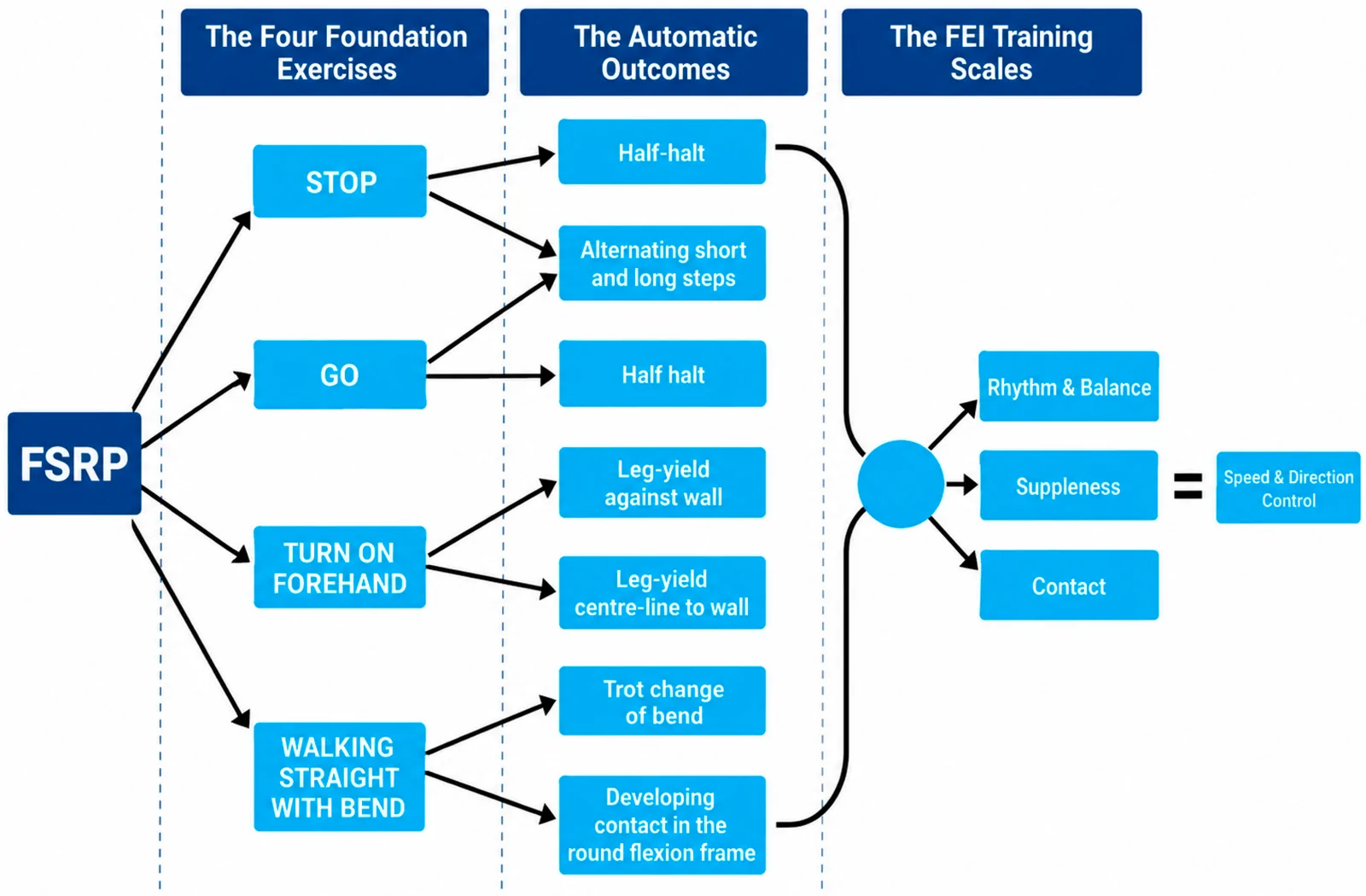
The development of the first three elements of the Fédération Equestre Internationale (FEI) training scales via the four foundational exercises and the six automatic outcome exercises of the Four-Step Riding Programme [51].

**Figure 3:**
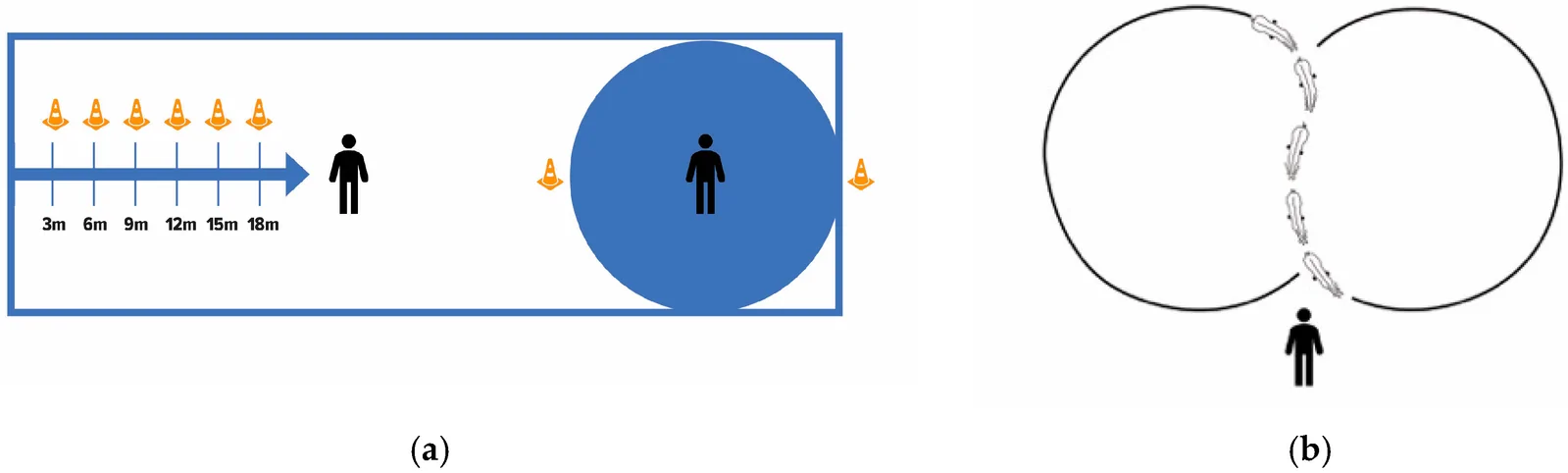
(**a**) Videographer position at the circle centre for most exercises and at the end of a 12 m line for the “walking straight with a bend” exercise. (**b**) Videographer position opposite the intersection of the two circles during the “change of bend at trot” exercise.

**Figure 4:**
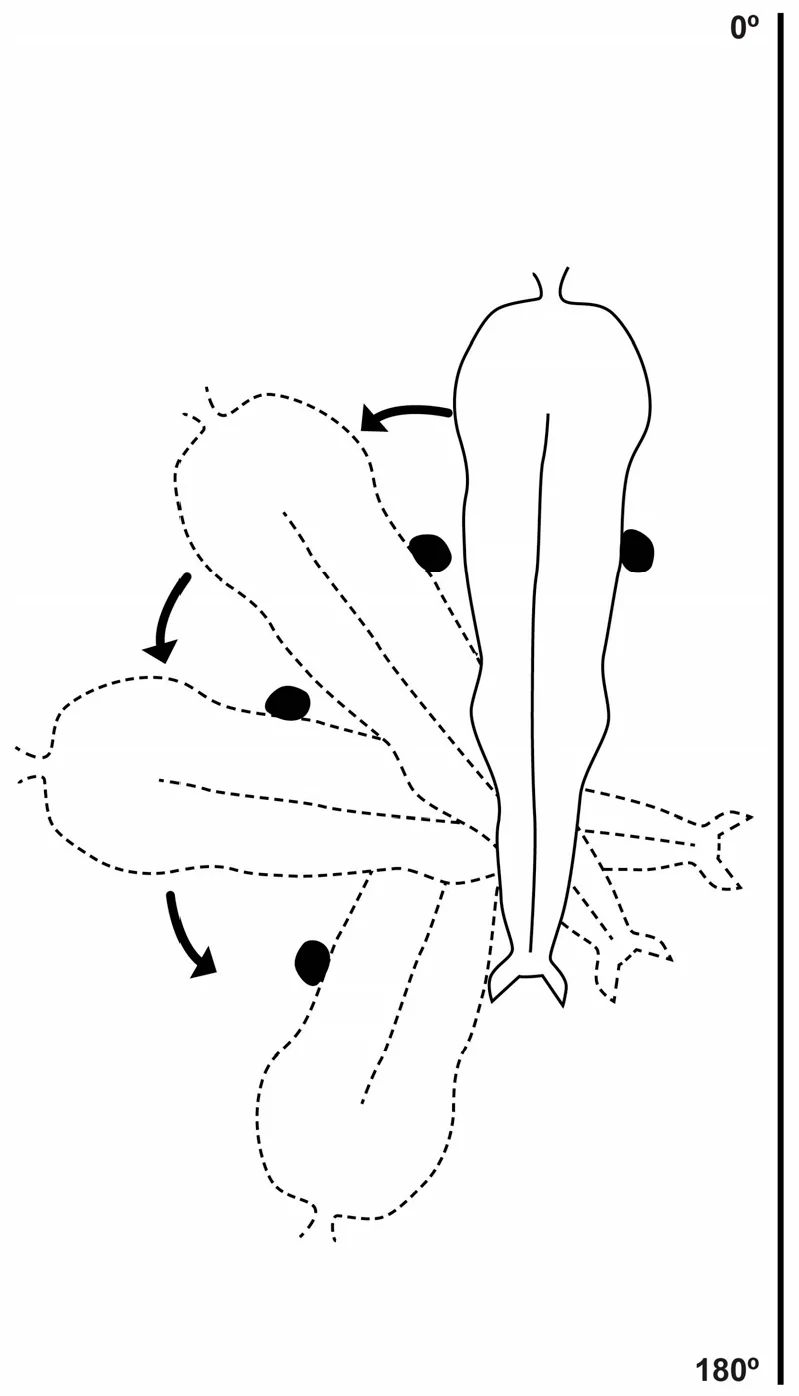
The turn on the forehand with the black spheres showing the rider’s leg pressure on the horse’s thorax, the static front legs of the horse, and the lateral steps of the hind leg. Reproduced with permission from K. Blignault, Four Steps to Riding Success, published by J.A. Allen, 2014 [19].

**Figure 5:**
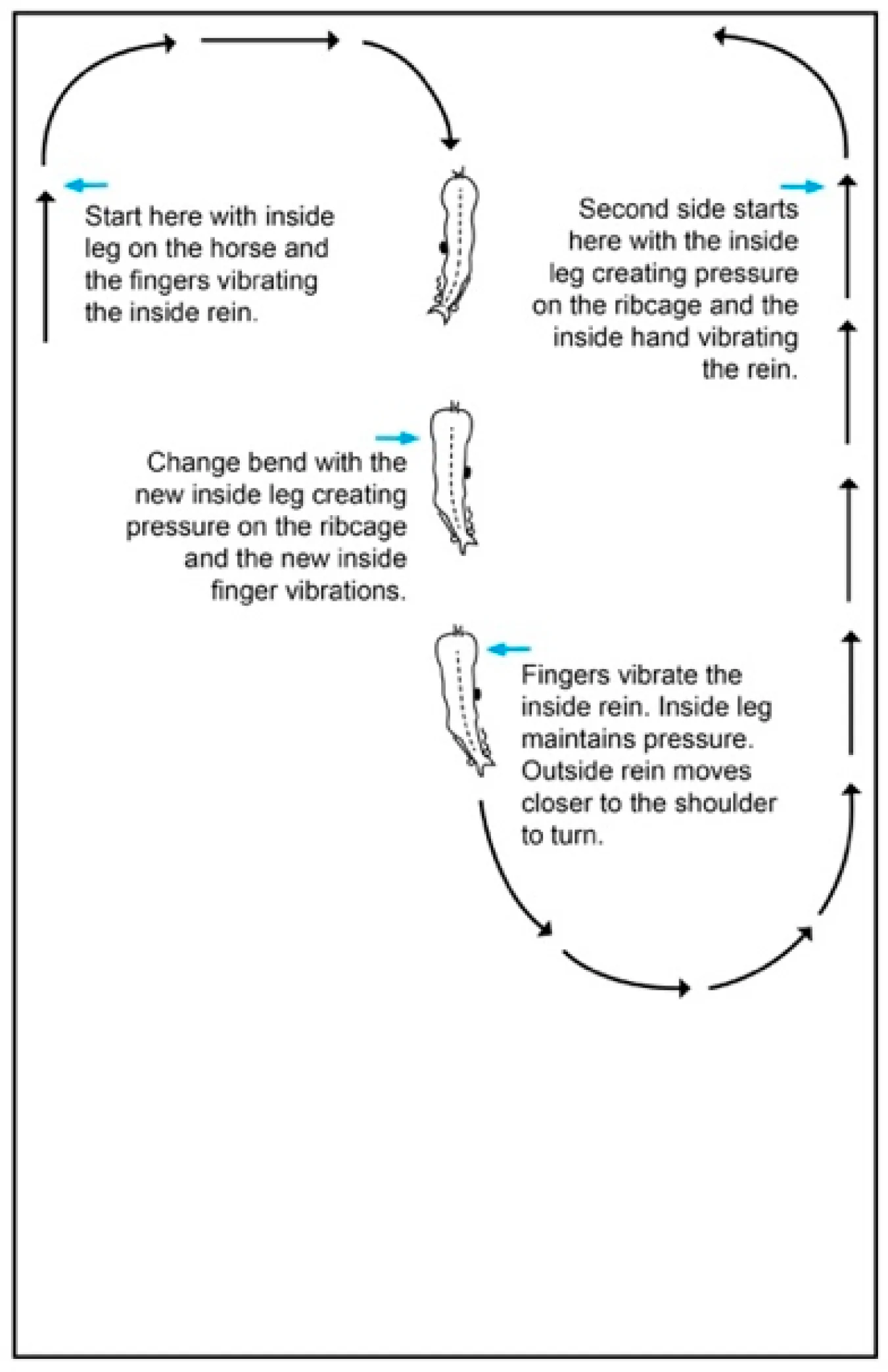
The walking straight with a bend exercise. Reproduced with permission from K. Blignault, Four Steps to Riding Success, published by J.A. Allen, 2014 [19].

**Figure 6:**
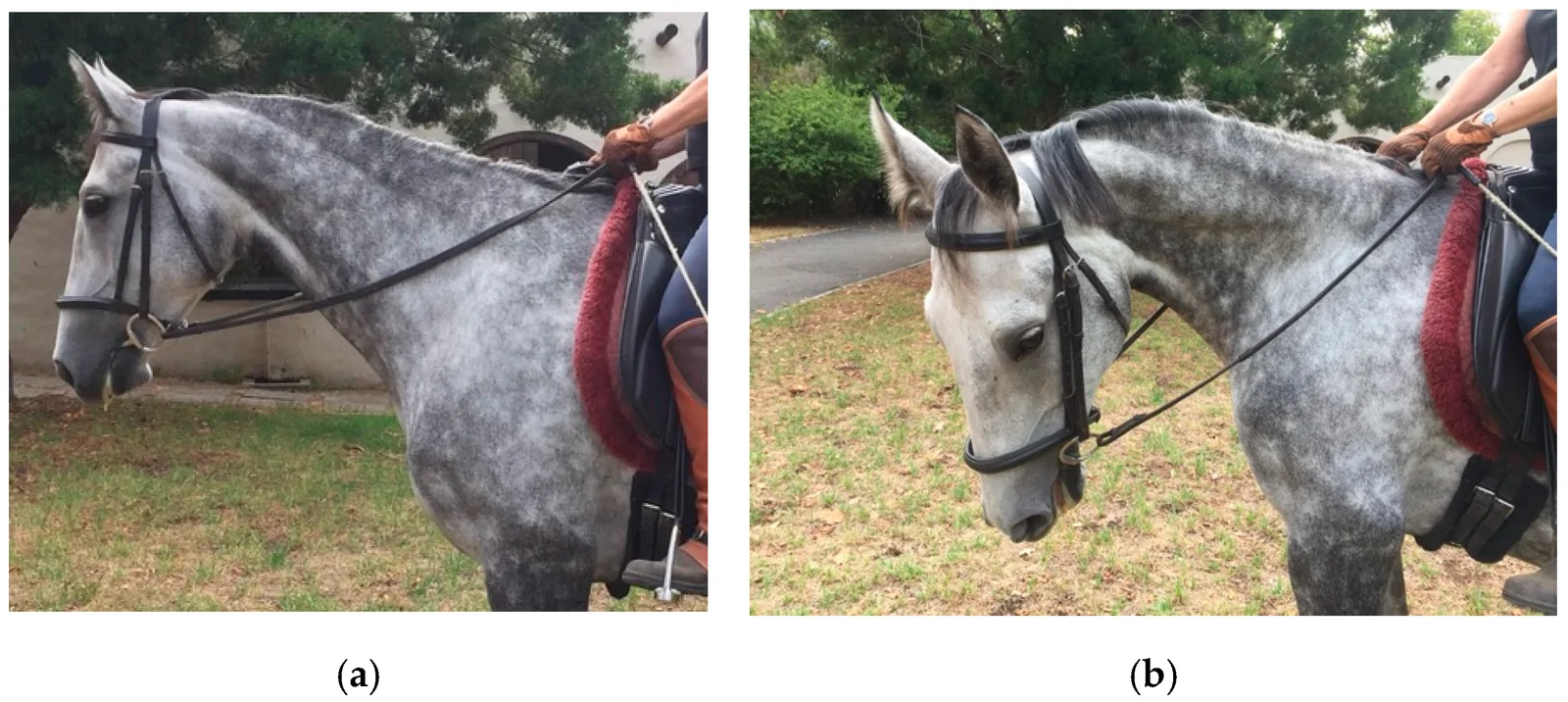
(**a**) Teaching the flexion frame of contact. The rider’s light pressure rein squeeze cuing the horse for lateral flexion (bend). (**b**) The horse responding with a clear lateral flexion together with flexion in which its nose takes on the vertical position of contact. It shows the instant release of rein pressure as the horse yields. Note the active inside neck muscle contraction with the resultant loose inside rein and straight outside rein evidencing the horse bending his own neck. (Photos from author’s collection).

**Figure 7:**
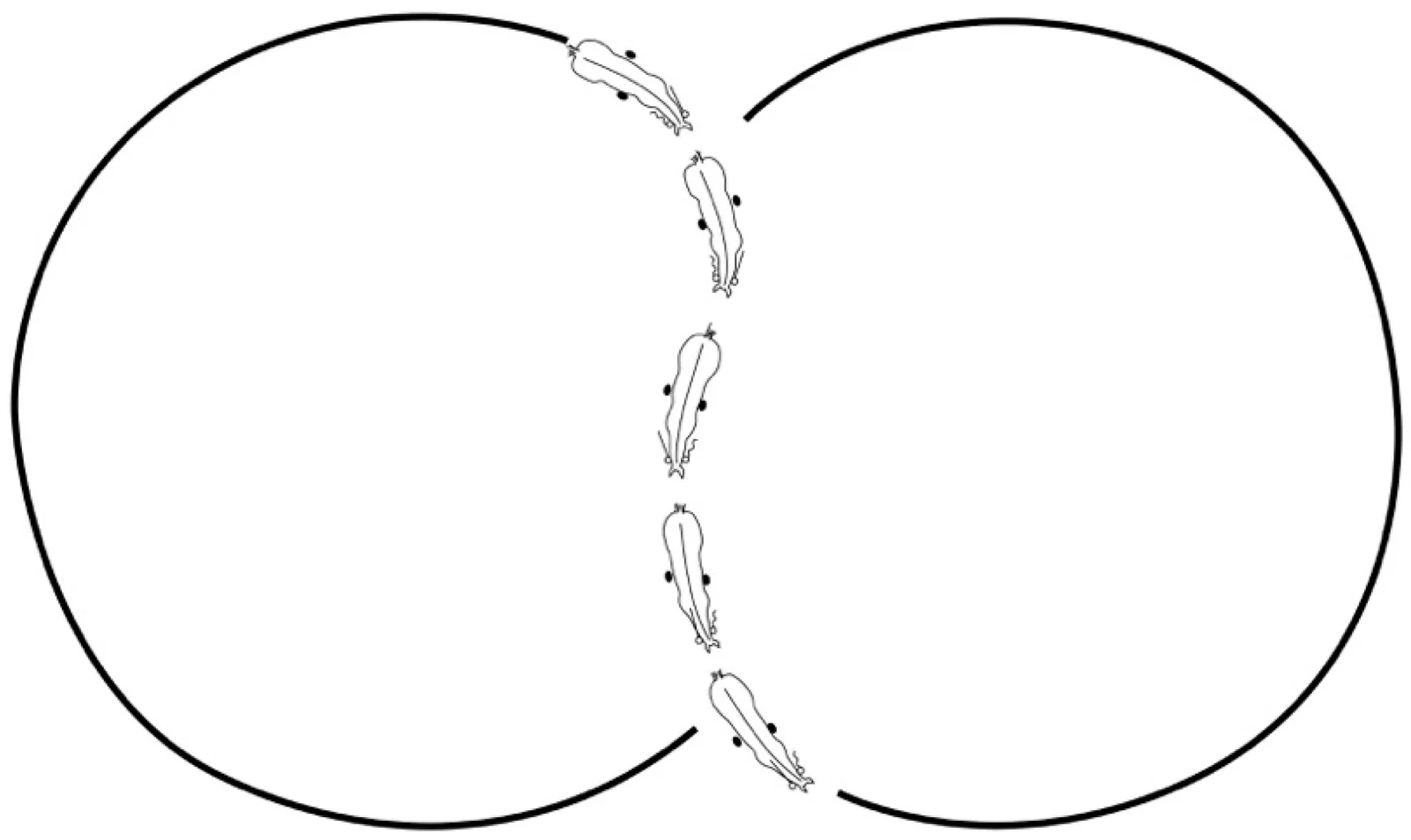
The change of bend at trot exercise. Reproduced with permission from K. Blignault, Four Steps to Riding Success, published by J.A. Allen, 2014 [19].

**Figure 8:**
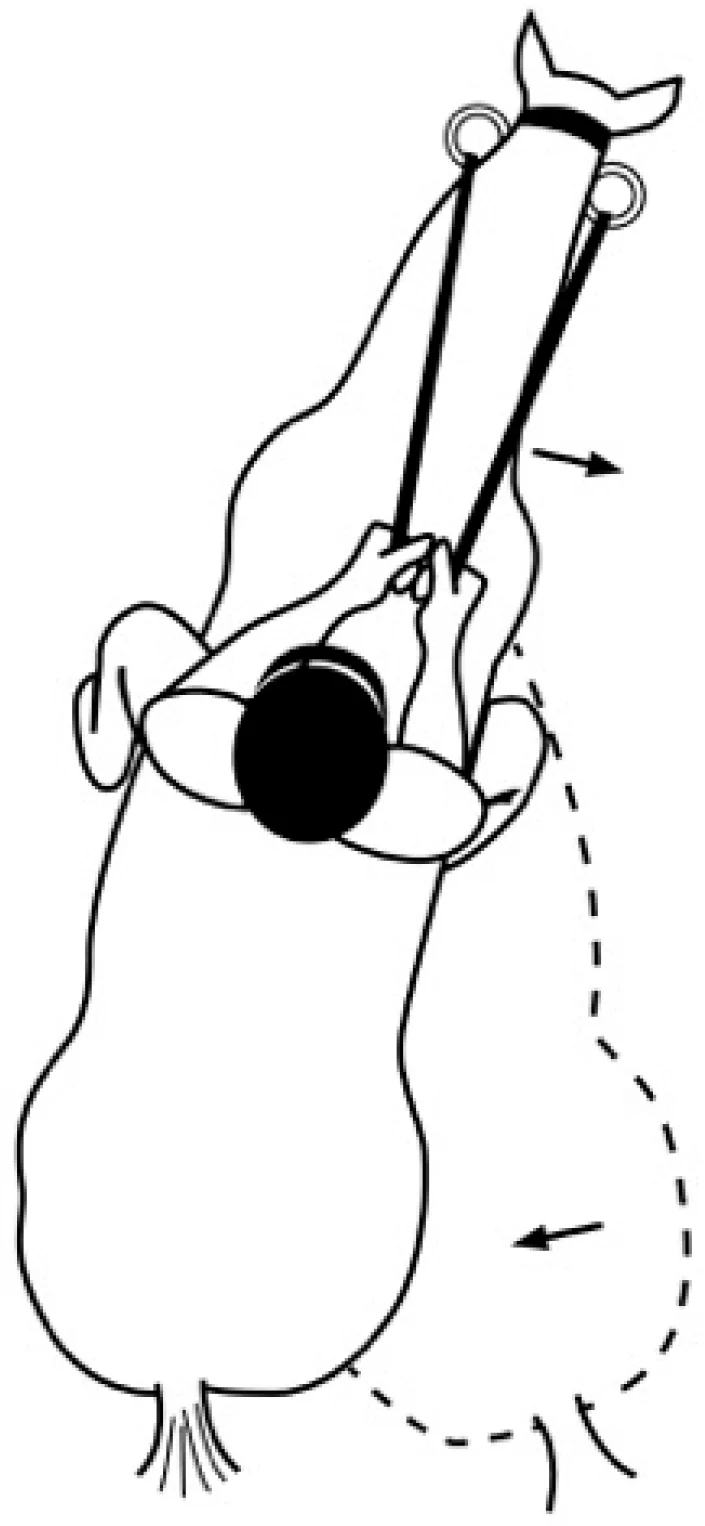
Controlling the angle of the hindquarters by influencing the shoulder movement.

**Figure 9:**
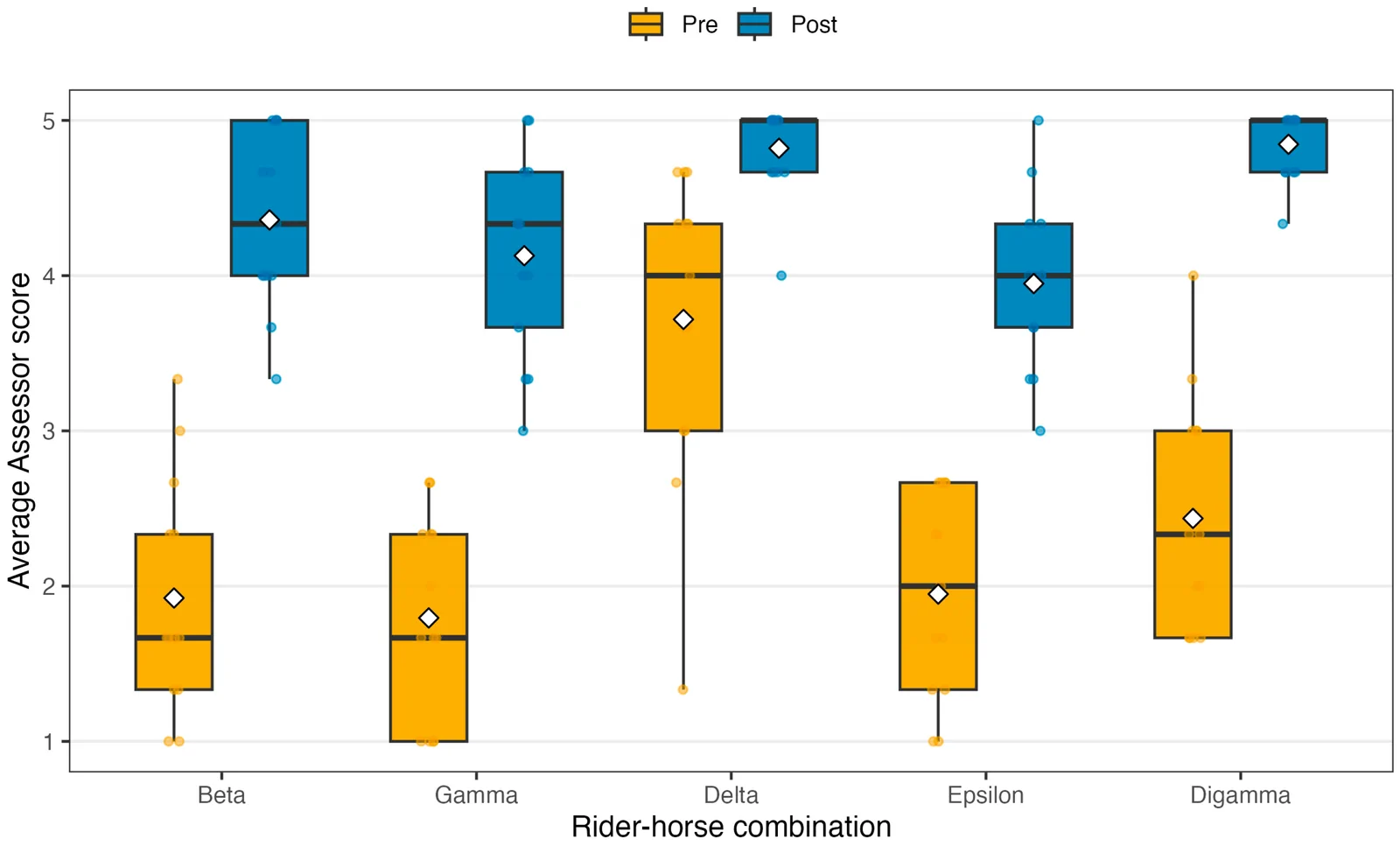
Pre- and post-intervention assessor-rated direction-control scores for each rider–horse dyad. Individual points represent the 13 direction-control variables, boxes show the interquartile range with the median as a horizontal line, and diamonds indicate the mean. Scores are on a 1 to 5 scale.

**Figure 10:**
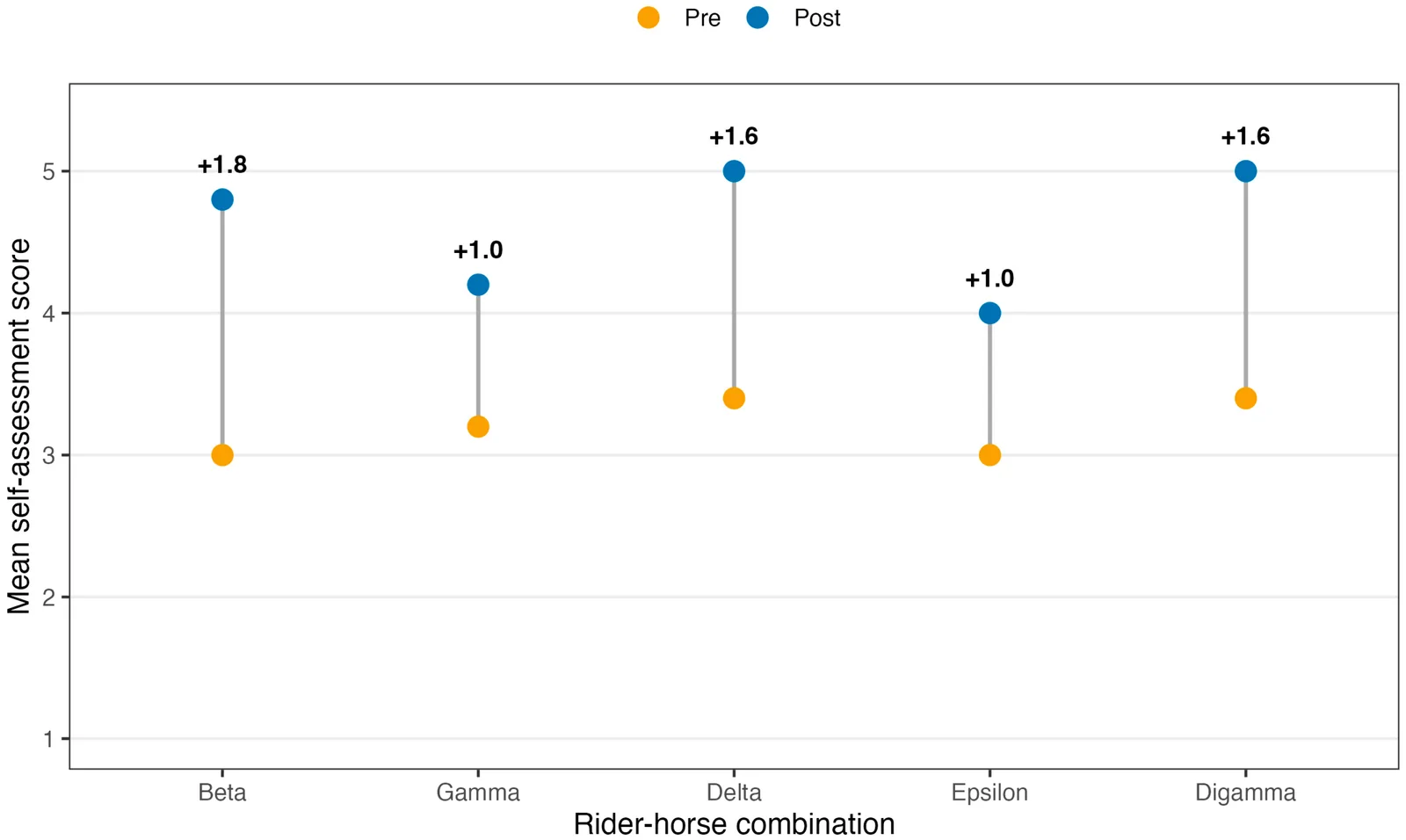
Mean participant self-assessment scores before and after the Four Step Riding Programme for each participant. Dots show the mean of the five self-assessed problem areas at each time point, and labels indicate the change in mean score from pre- to post-intervention. Scores are on a scale of 1 to 5.

**Figure 11:**
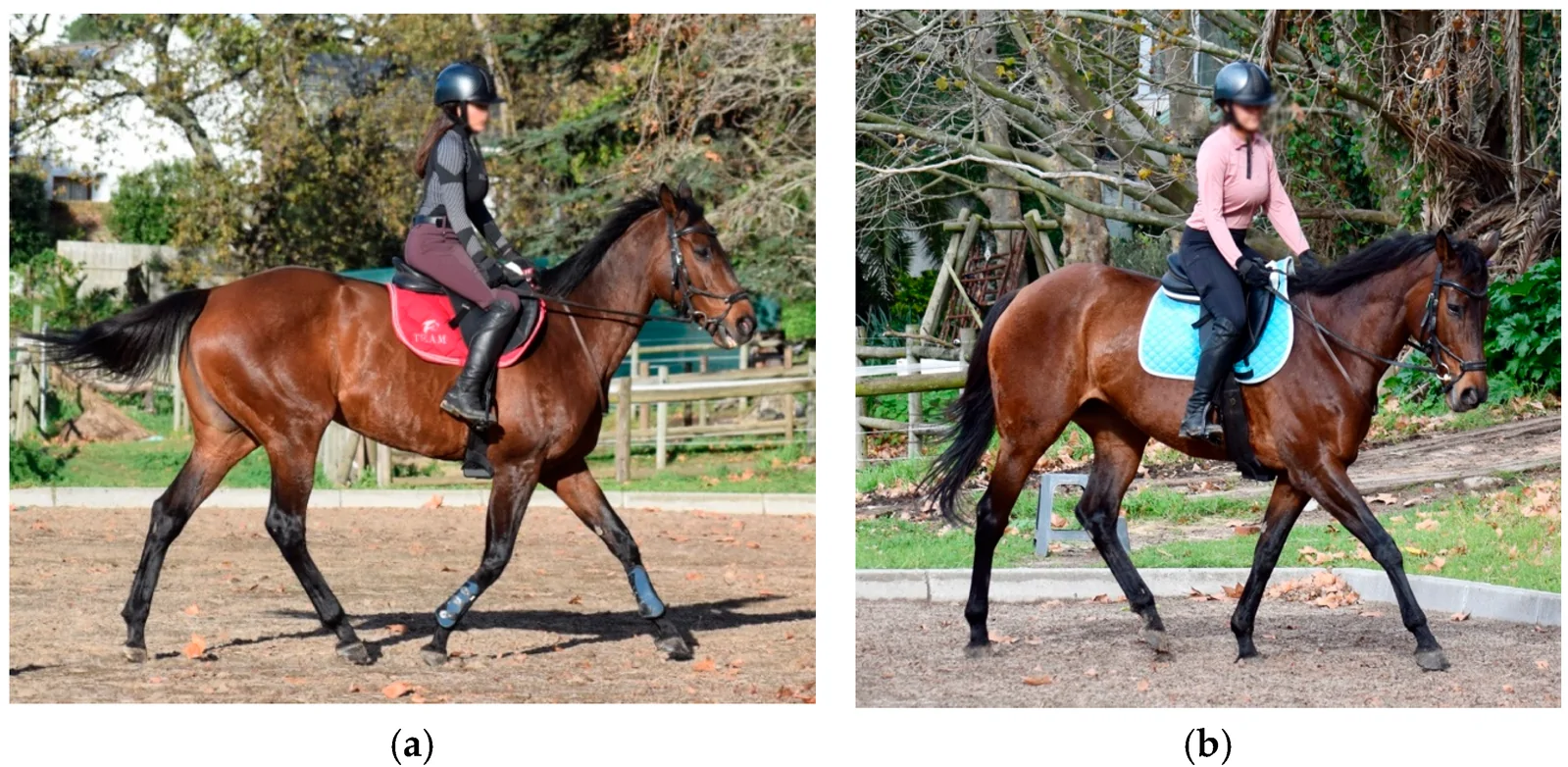
(**a**) Beta before the intervention in a right circle showing a hollow frame lacking suppleness. (**b**) Beta after the intervention showing improved flexion, suppleness, and shoulder control.

**Figure 12:**
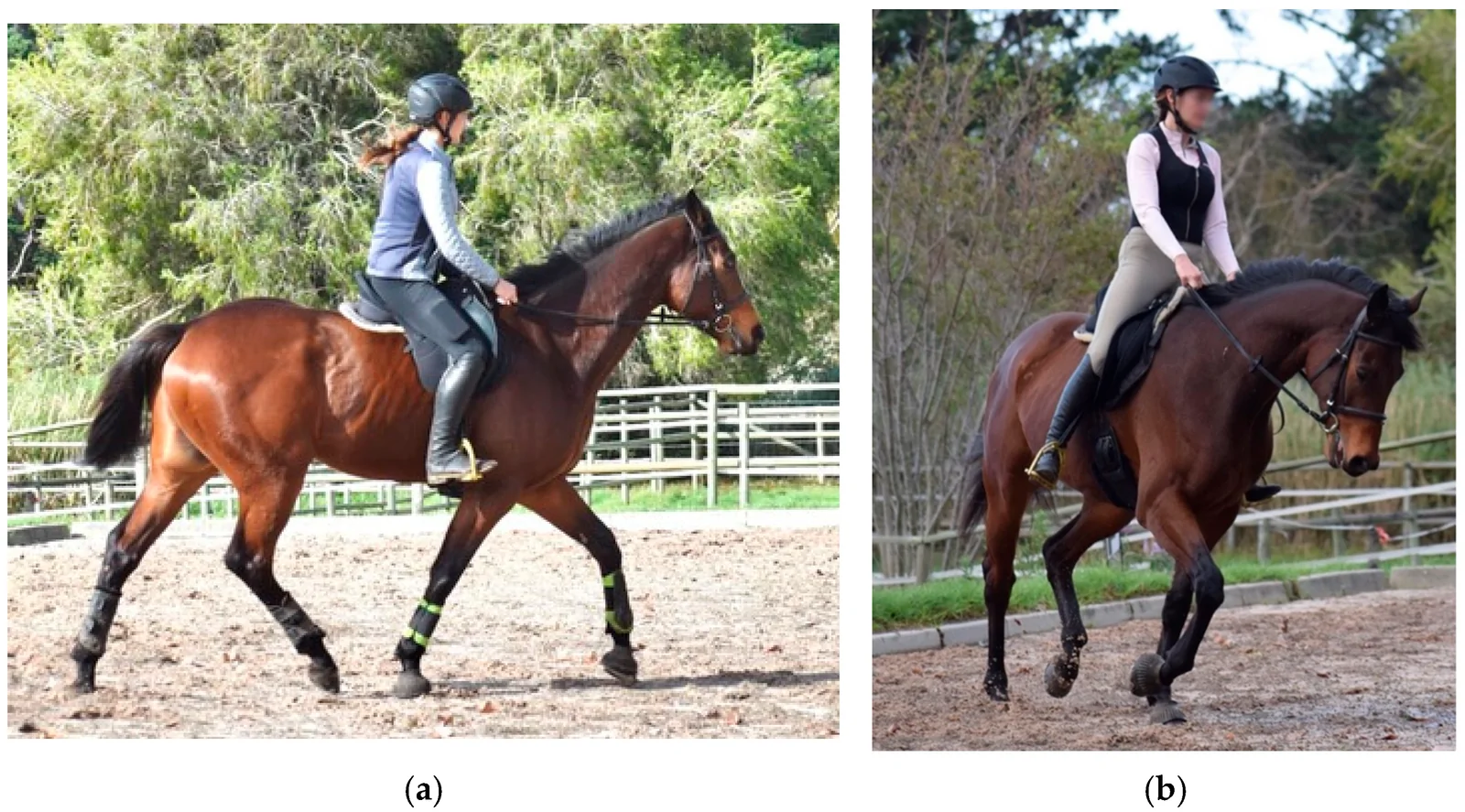
(**a**) Gamma before the intervention in a left circle shows a high head carriage, hollow frame, and a lack of suppleness. (**b**) Gamma after the intervention showing a round frame, clear bend, and improved shoulder control.

**Figure 13:**
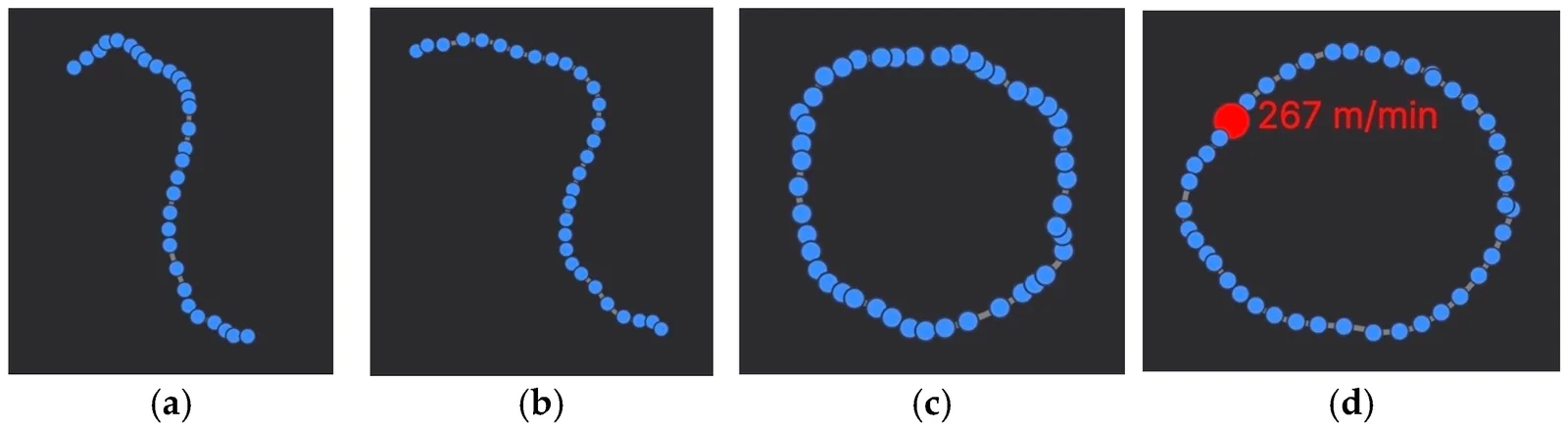
(**a**) Pre-intervention right-to-left change of bend exercise by Gamma showing irregular rhythm and balance difficulties. (**b**) The post-intervention image shows improved rhythm, balance, and shoulder control. (**c**) Gamma’s pre-intervention left circle showing irregular rhythm and balance. (**d**) The post-intervention image shows improved shape, shoulder control, rhythm and balance.

**Figure 14:**
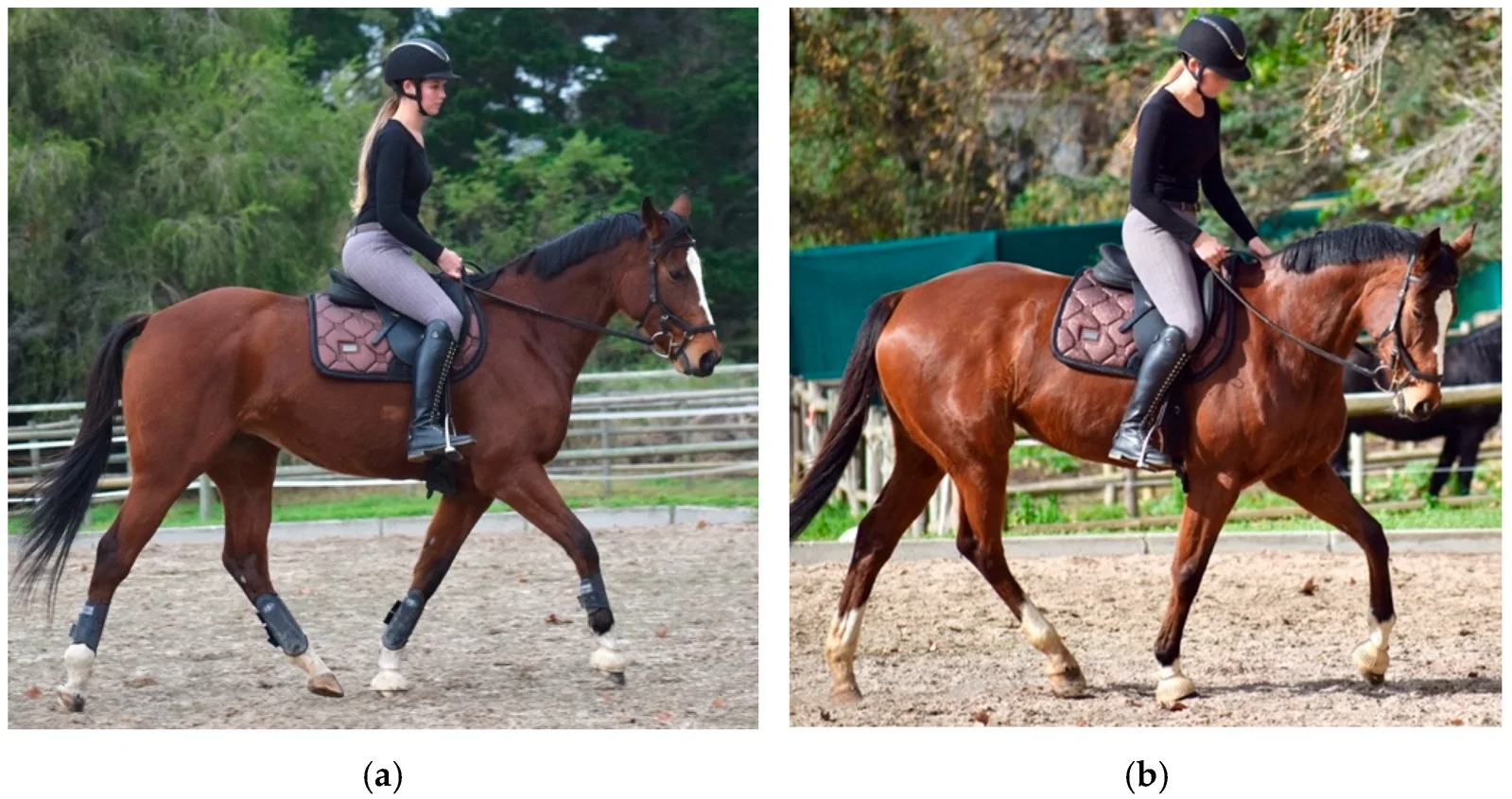
(**a**) Delta before the intervention in the right circle, showing a hollow frame lacking suppleness. (**b**) Delta after the intervention shows visible contraction in the inside neck muscles, indicating a clear improvement in lateral suppleness, flexion and shoulder control. The rider visually reinforces the correct feel by observing the horse’s head and neck position.

**Figure 15:**
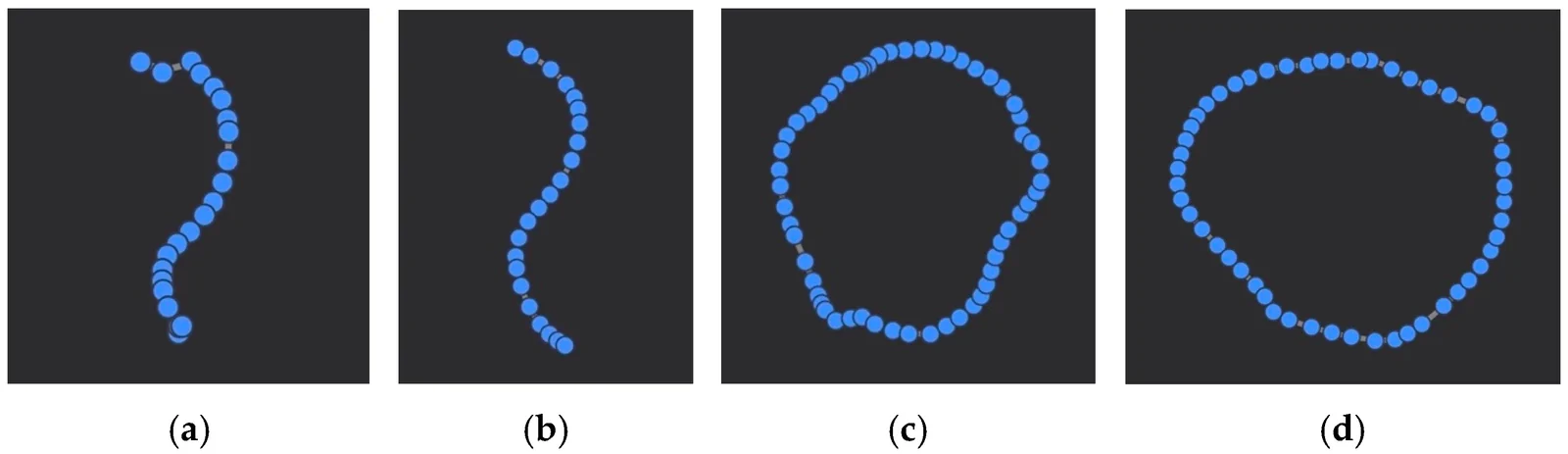
(**a**) Pre-intervention satellite image of Delta’s right-to-left change of bend exercise, uneven rhythm, and balance. (**b**) After the intervention, Delta demonstrated improved rhythm, balance, and shoulder control. (**c**) Delta’s pre-intervention left circle showing a lack of shoulder control and rhythm. (**d**) After the intervention, Delta showed a clear improvement in circle shape, shoulder control, rhythm and balance.

**Figure 16:**
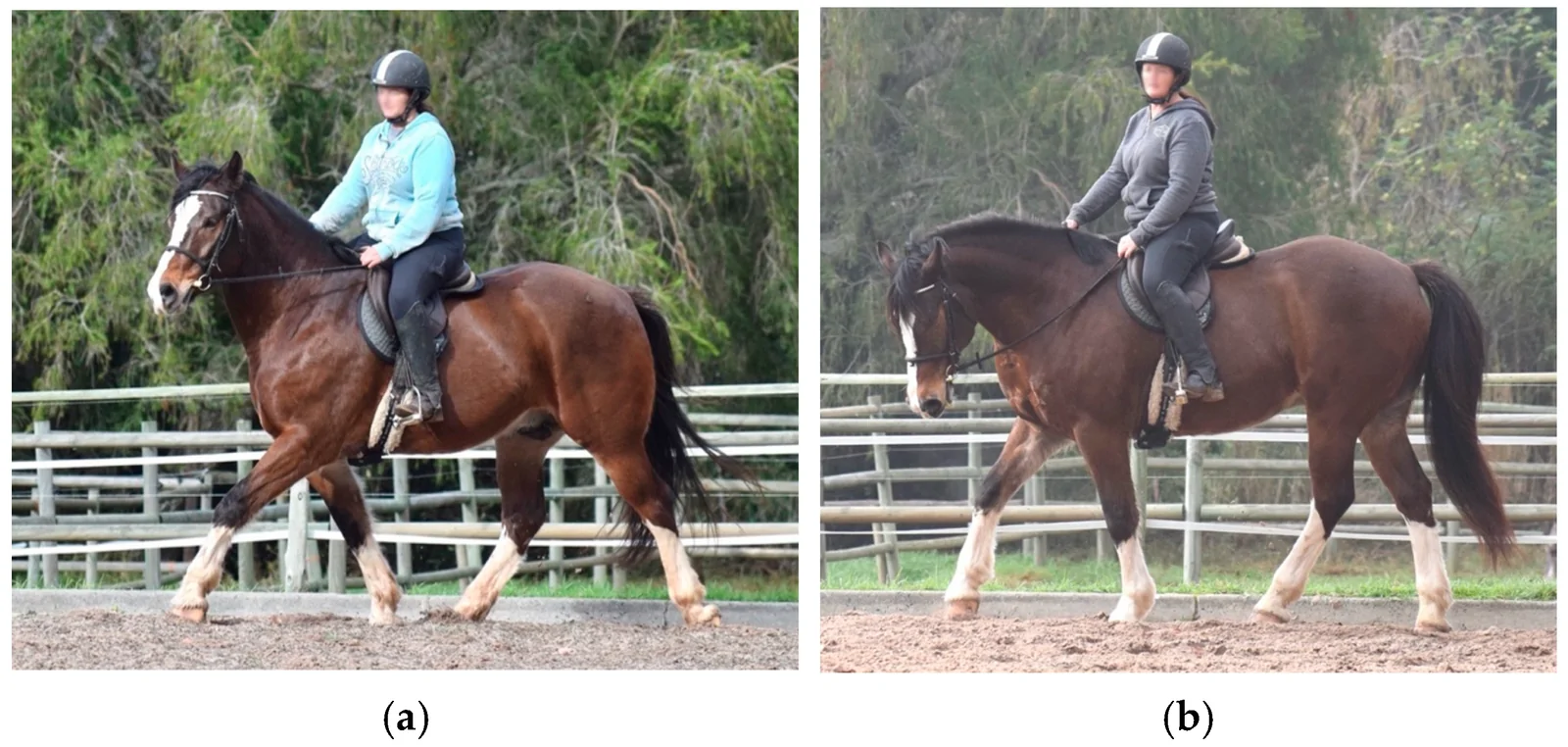
(**a**) Epsilon’s pre-intervention left circle showing a hollow frame lacking longitudinal and lateral suppleness or bend. The rider is pulling the rein to turn. (**b**) Post-intervention photograph showing visible inside neck muscle contraction with a soft inside rein indicating clear improvement in lateral and longitudinal flexion suppleness and shoulder control with the outside rein.

**Figure 17:**
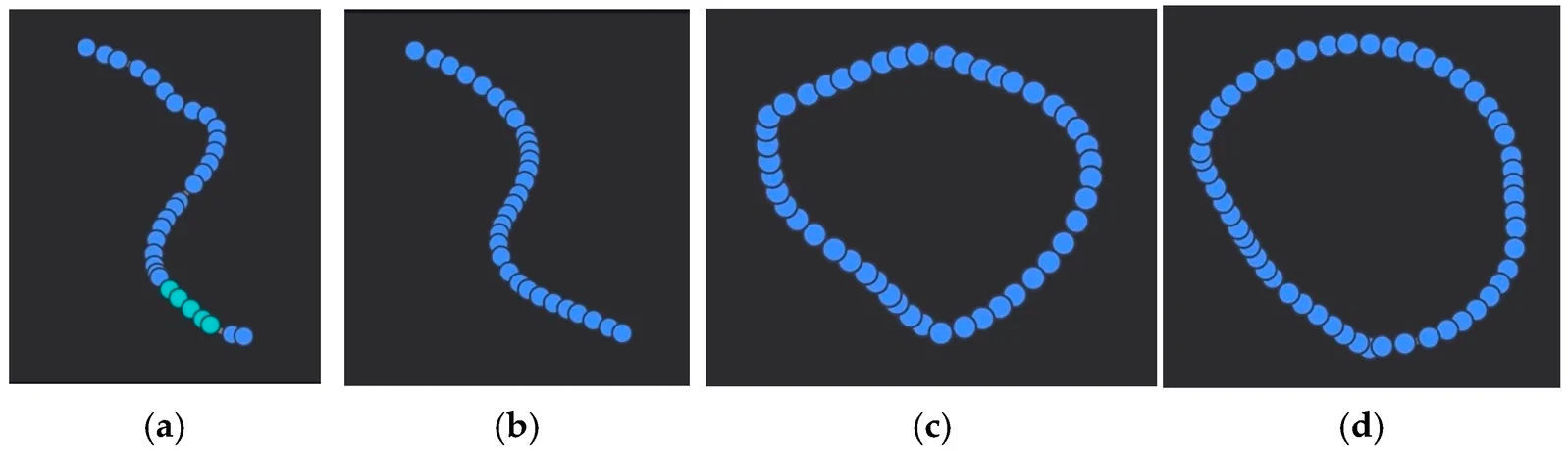
(**a**) Pre-intervention satellite image of Epsilon’s right-to-left change of bend exercise showing rhythm and balance difficulties with lack of shoulder control. (**b**) After the intervention, Epsilon showed improved rhythm and balance. (**c**) Epsilon’s pre-intervention right circle indicates a lack of shoulder control, rhythm and balance. (**d**) After the intervention, Epsilon showed clear improvement in circle shape, shoulder control, rhythm, and balance.

**Figure 18:**
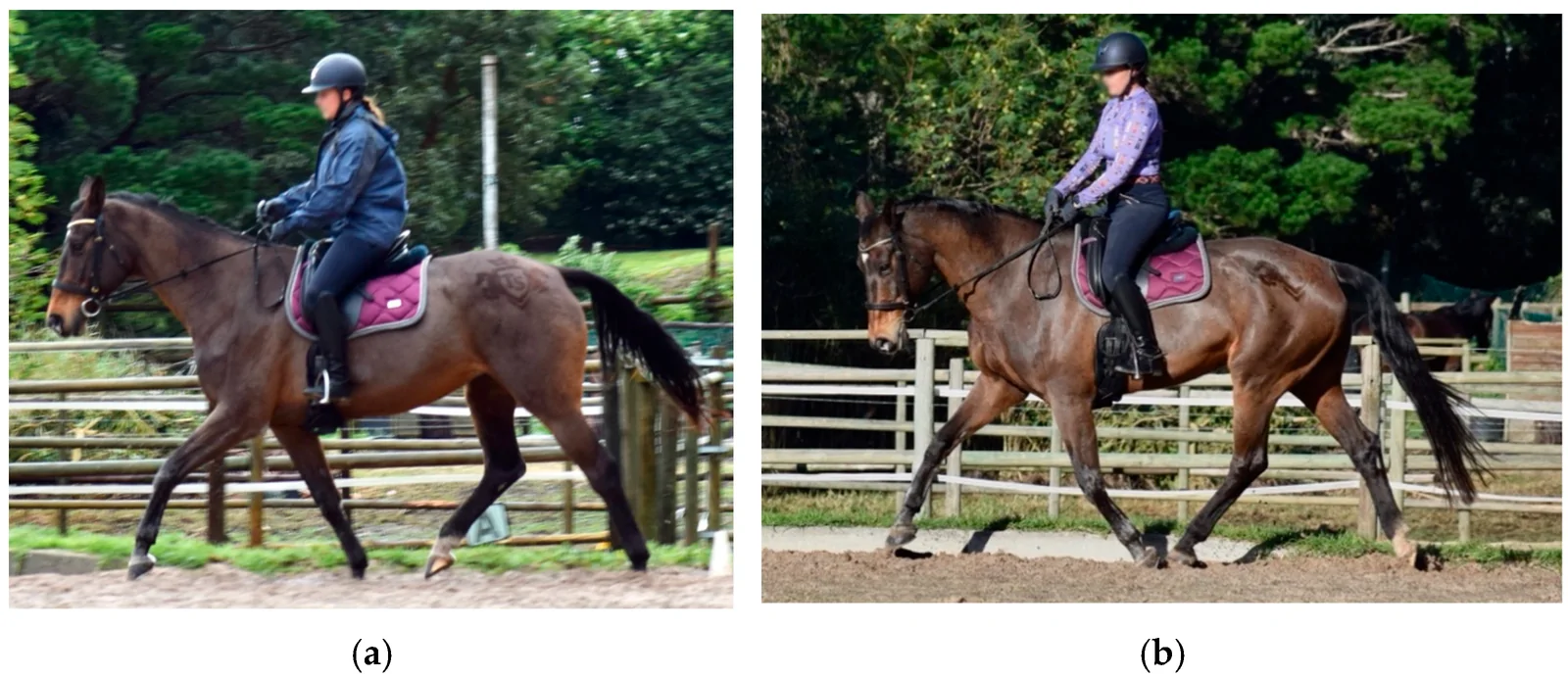
(**a**) Digamma’s pre-intervention left circle showed a hollow frame lacking suppleness or bend. The pulling left arm and the horse’s straight neck with no muscle definition show the lack of bend into the corner. The hindquarter appears to lack engagement. (**b**) The post-intervention image shows clear improvement in lateral (bend) and longitudinal suppleness in the flexion frame of contact with improved shoulder control. The hindquarters appear to be more engaged with more propulsion.

**Figure 19:**
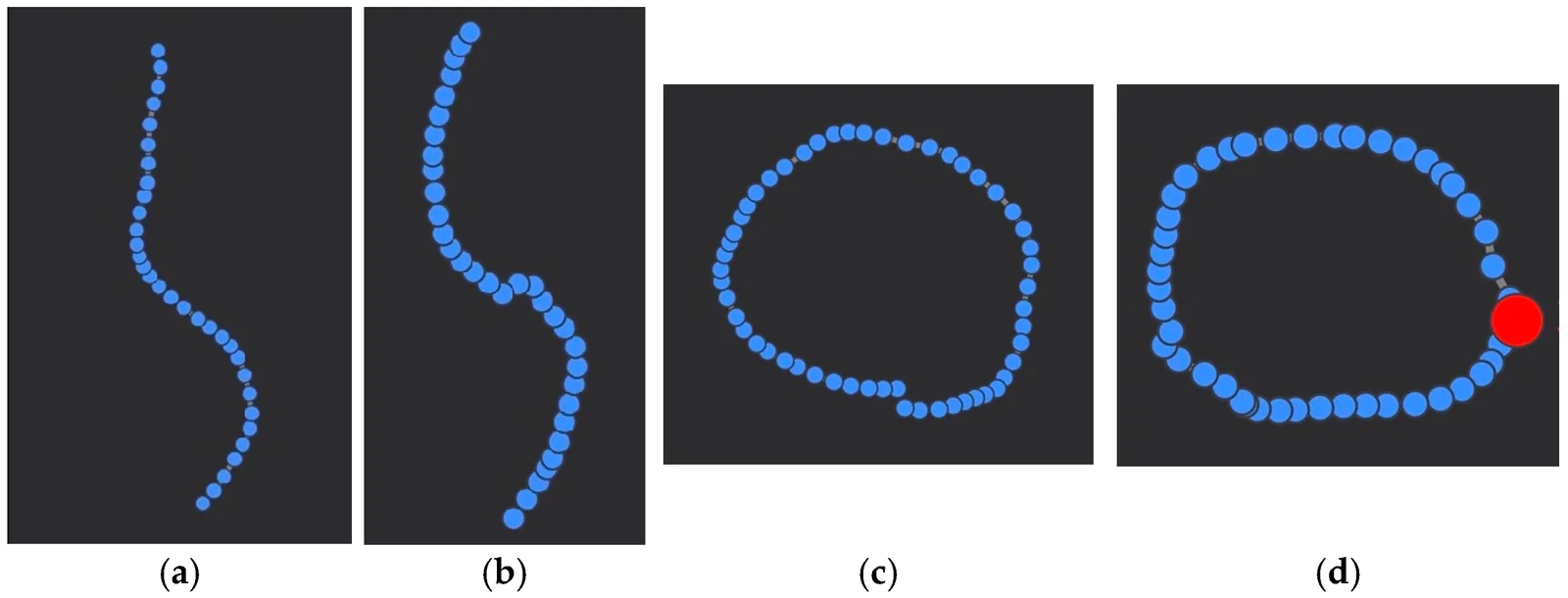
(**a**) Pre-intervention satellite image of Digamma’s right-to-left change of bend exercise showing rhythm and balance difficulties with lack of shoulder control and lack of change of bend. (**b**) Rhythm and balance improved after the intervention. The change in bend is evident as a side step, which is a clear indication of improved shoulder control. (**c**) Digamma’s pre-intervention right circle showing a lack of shoulder control, rhythm and balance. (**d**) Rhythm clearly improved after the intervention.

**Table 1:** Inclusion criteria for rider–horse dyads in the study.

Riders	Horses
More than five years of riding experience	Fit, healthy, and sound horses only
Regular weekly riding lessons for at least six months	Aged up to and including 16 years
Adults aged 20–49 years	Properly fitted saddles
Self-reported communication or training issues with their horses	

**Table 2:** Baseline characteristics of the five horse–rider dyads.

Horse–Rider Dyad	Rider Age (Years)	Horse Age (Years)	Years of Riding	Years of Riding Lessons	Years with Current Horse
Beta	21	13	12	12	2
Delta	28	7	20	18	4
Digamma	36	16	26	26	3
Epsilon	46	9	42	40	6
Gamma	21	4	14	14	0.7

**Table 3:** Step-by-step explanation of the Four-Step Riding Programme (FSRP).

Step	Explanation	Standardisation
Pre-training assessment.	The instructor evaluates the horse-rider dyad for riding communication problems.	Standard for all participants.
Introductory lecture.	A 30 min lecture on equine biomechanics and anatomy, body language, and methods by which horses learn.	Standard for all participants.
Instructor teaches the horse to respond to clicker training.	Horse touches the cone, mark with a click, reward the horse with a treat. Used to mark and reward correct steps when teaching the horse and rider the turn on the forehand and the leg-yield movements.	All horses learned this within three trials.
Lesson 1: FSRP four basic exercises taught.	Refer to Table 4 for a summary of the exercises.	All participants were taught these exercises in the same sequence. Multi-modal motor learning techniques were standard for all riders.
Lesson 2–4: Correction of FSRP basic exercise taught in Lesson 1.	Refer to Table 4 for a summary of the exercises.	All participants were taught these exercises in the same sequence. Multi-modal motor learning techniques were standard for all riders.
Lesson 3: Yielding in a round flexion frame (contact) taught, correction in Lesson 4.	Refer to Table 4 for a summary of the exercises.	All participants were taught these exercises in the same sequence. Multi-modal motor learning techniques were standard for all riders.

**Table 5:** Intraclass correlation coefficients for agreement between assessors for pre-intervention and post-intervention for direction control.

	Pre-Intervention		Post-Intervention	
	ICC Value	*p*-Value	ICC Value	*p*-Value *
Beta	0.89 (good)	0.00	0.66 (moderate)	0.01
Gamma	−0.22 (poor)	0.63	0.78 (good)	0.0008
Delta	0.81 (good)	0.0003	0.60 (moderate)	0.03
Epsilon	0.40 (poor)	0.14	0.67 (moderate)	0.0098
Digamma	0.51 (moderate)	0.07	0.2350 (poor)	0.33

* *p*-values < 0.05 indicate significance at a 5% level.

**Table 6:** Paired Wilcoxon signed-rank test results and effect sizes for average assessor-rated direction-control scores.

Rider–Horse Dyad	Pre-Intervention Average Score	Post-Intervention Average Score	Difference (Post Minus Pre)	*p*-Value	Effect Size (r)
Beta	1.92	4.36	2.44	0.0002	0.884
Gamma	1.79	4.13	2.33	0.0002	0.883
Delta	3.72	4.82	1.1	0.001	0.857
Epsilon	1.95	3.95	2.00	0.0002	0.883
Digamma	2.44	4.85	2.41	0.0002	0.885

**Table 7:** Paired Wilcoxon signed-rank test and effect sizes for participant self-assessment scores.

Rider	Wilcoxon Test *p*-Values	Effect Size	Magnitude
Beta	0.03	0.91	Large
Gamma	0.09	0.76	Large
Delta	0.03	0.93	Large
Epsilon	0.04	0.87	Large
Digamma	0.03	0.93	Large

## Data Availability

The data supporting the findings are available at https://researchdata.up.ac.za/.

## Supplementary Materials

The following supporting information can be downloaded at: https://www.mdpi.com/article/10.3390/ani16203170/s1. Section S1: explaining the exercise in more detail; Figure S1: explaining the Multi-Modal Motor Learning Techniques; Figure S2: explaining the Multi-Modal Motor Learning feedback; Figure S3: explaining the assessors’ instruction protocol; Figure S4: summarising the assessor scoring criteria; Figure S5: Summarising the variables and exercises used in the FSRP; Table S1: describing the horse and rider demographics; and Table S2: describing the deviations implemented during the use of the Multi-Modal Motor Learning Techniques and feedback. Ref. [58] is cited in Supplementary Materials.

## Abbreviations

FSRP (Four-Step Riding Program); MMLT (multi-modal motor learning technique); ICC (intraclass correlation coefficient).
