# Supplementary material for: Teaching Direction Control in Horse Riding: A Pre–Post Intervention Proof-of-Concept Study Using Multi-Modal Motor Learning Techniques
Source: Animals (Basel). 2026 Oct 9;16(20):3170. doi: 10.3390/ani16203170 (PMC13700739; doi:10.3390/ani16203170)
Supplement: Supplementary file 1 [file animals-16-03170-s001.zip › Section S1 Exercises.pdf]

Direction Control Section S1 – The exercises

1. **The turn on the forehand**

This is the first exercise to teach shoulder control, which also controls the horse's forefeet. It illustrates the phenomenon of the horse taking lateral steps to turn and teaches the rider how to cue the horse to take lateral steps with his hind legs. The execution of the lateral stepping has to be precise because this movement establishes the foundational skills for subsequent lateral exercises. The exercise is done at a halt against the arena barrier. The horses' forelimbs remain static while the hind limbs take individual lateral steps around his forelimbs in a half circle (180º) until the horse faces the opposite direction. The rider's hands are used together to prevent unelicited forward and backward movements and lateral shoulder movements. Two common mirror movements associated with this exercise often confuse horses and lead to slow learning. These are, the inadvertent application of simultaneous bilateral leg pressure which creates opposing instructions to the horse, instead of single leg pressure. To correct this problem, the rider's inside-leg has to be removed off the horse. The second mirror movement is an inadvertent pull back of the rein on the ipsi-lateral side of the rider's weaker leg, and simultaneous release of the opposite rein/arm which moves forwards with the hand opening and allows the rein to slip [19]. To correct this mirror movement, the rider's hands are constrained by the rider placing one thumb in the palm of the other hand.

The MMLT incorporated in this exercise included blocked practice; visual demonstration; verbal explanation and feedback; tactile pressure; kinaesthetic and proprioceptive feedback; guided movement; a constraints-led approach and external focus [51]. This exercise typically needs to be repeated numerous times for riders to acquire the complex coordinated movement patterns between the legs, hands and body which prevent horse confusion. Whereas horses generally acquire this movement in a single training session provided the cues are accurately applied with good timing.

2. Walking straight with a bend

A controlled degree of lateral flexion of the horse's head and neck is required when preparing the horse to negotiate circles, corners, turns and lateral movements. This exercise provides the foundation for the leg-yield and shoulder-in. The objective of the walking straight while maintaining lateral flexion is to develop the rider's ability to control the horse's shoulders while preserving lateral flexion of the head and neck through the turn and simultaneously maintaining the horse's balance. Two preliminary exercises demonstrated that the inside rein can only decrease the size of a circle, while the outside rein, moving closer to, or further away from the horse's shoulders, determines the size of the circle or turn. It is the fundamental exercise which teaches direction control and concurrently teaches riders to use the outside rein to turn.

The horse walks on a straight line up the centre line of the arena. The rider's inside leg, positioned on the horse's thorax at the girth area, is used to apply pressure to the horse's ribcage while simultaneous light finger vibrations on the inside rein create and maintain the slight lateral flexion. The rider's outside leg remains slightly off the horse's body. The rider cues the horse to change the bend by changing leg position as well as rein cues. Once the inside bend is established and maintained on the straight line and without an anticipatory turn, the outside rein is moved closer to the horse's shoulders to cue the horse to start the turn towards the track. The size of the turn is maintained with the outside rein. Once the horse reaches the track, the slight inside bend is maintained while walking on the track until he is ready to turn up the centre line. The outside rein is then moved closer to the horse's shoulders

to cue him to turn up the centre line. The exercise is then repeated with a change to the opposite direction [19].

Substantial practice is required to integrate the sensory-motor processes needed for the complex coordination skills to perform this exercise effectively. The horses are dependent on the rider's acquisition of the coordinated motor skills needed for clear body-language cues.

The MMLT incorporated to teach the appropriate motor skills were verbal explanation together with visual demonstration; tactile and kinaesthetic input for the rider to experience the feel of opening the outside rein the vibrating inside rein and how to create the appropriate pressure with the inside-leg. Verbal cueing; blocked practice and verbal feedback on knowledge of performance and knowledge of results were provided [51].

3. Yielding in a round flexion frame – contact

The round flexion contact frame should emerge as an automatic response to the use of light finger vibrations for the development of lateral flexion in the walking straight with a bend exercise. Where this natural response fails to develop, the horses are taught to yield to rein pressure first. The principle underlying the development of responsiveness to light leg pressure namely, negative reinforcement of the application of pressure, the horse's appropriate response, and the immediate cessation of pressure, is similarly applied to teaching the horse to yield to rein pressure. Entrenched defensive mouth resistances often develop when riders are taught the common method of simultaneous rein and leg pressure cues which provide the horse with confusing contradictory directions of stop and go [58]. Therefore, light finger vibrations are applied and when the horse does not respond, strong enough pressure such as squeezing with the whole hand and releasing it as the horse yields, is applied. Once the horse reliably yields to one rein pressure, the same procedure is repeated on the opposite side until the horse yields instantly to light finger vibrations. Adding positive reinforcement further cements the correct response from the horse [19]. This is practiced at a halt and once established it is practiced at the walk and then consolidated at the trot as explained in the next exercise. Intermittent light finger pressure reminds the horse to yield throughout the 20m walk and trot circles.

It is important to distinguish between continuous alternating rein pressures which pull the horse's head from side to side and is commonly referred to as 'sawing, and the horse yielding to light rein squeezes. The need to continue with these pulling actions indicate that the horse has not appropriately responded to the strongly applied rein pressures. In contrast, correctly timed and light finger pressure should produce an authentic yielding to the cue rather than having its head mechanically pulled from side to side. Therefore, no pulling on the reins is permitted in this exercise.

The MMLT incorporated to teach the round flexion frame of contact included guided movement; visual demonstration; verbal explanation; tactile, kinaesthetic proprioception modalities and a constraint-led approach in which the riders closed their eyes to feel the contact.[51].

4. Trotting two 20m circles with a change of bend in between the two circles for shoulder control and bend

The primary objective of this exercise is to consolidate the rider's coordination ability to precisely control the horse's shoulders while changing bend at the faster gait of trot without the horse losing balance and turning prematurely. The movement is performed by riding two interchanging circles, which resemble a figure of 8 pattern and covers the width of the arena. This exercise is performed in both directions with the horse changing its bend during the transition from one circle to the other to ensure the development of equal suppleness. It is performed using the same hand, arm, body and leg cues which are used for the walking straight with a bend exercise. A half-halt is employed a few strides before the bend change cues to reduce the speed and provide the rider with more time to modify the body, leg and rein cues required to establish the change of bend to the new direction.

The MMLT incorporated to teach the coordinated motor skills included visual demonstration together with verbal explanation; guided movement; visual and verbal cueing; visualisation and mental imagery techniques and kinaesthetic, tactile and proprioception input [51].

5. The leg-yield with the horse's head to the wall for shoulder control

The object of the leg-yield is to teach riders to control the horse's shoulders while taking lateral steps with all four limbs [19]. Two mirror movements are commonly associated with this exercise. The non-dominant arm copies the ipsilateral leg action and both legs inadvertently apply simultaneous pressure whereas the outside leg should not apply pressure.

The leg-yield is performed with the horse positioned at an approximate 45° angle relative to the arena barrier. With both hands held together, the rider is taught to apply pressure with the inside-leg to the horse's thorax behind the girth area thereby eliciting the required lateral hind leg steps. Concurrently, the rider is taught how to maintain a consistent body angle by regulating the horse's shoulder movements throughout the leg yield.

Due to the equine quadruped's relatively rigid vertebral column, lateral displacement of the shoulders towards one side results in corresponding displacement of the hindquarters towards the opposite side. Consequently, when the angle between the horse and the arena barrier becomes too large, the rider is instructed to move both hands minimally in the direction of the horse's movement. Conversely, when the angle becomes too small, both hands are moved minimally towards the side of the leg applying the pressure. These actions reduce the need for excessive leg pressure to maintain the desired angle. Through trial-and-error learning, the rider develops the ability to determine the precise magnitude and lateral hand and arm movements required to maintain a consistent angle relative to the barrier.

The MMLT incorporated to acquire the motor skills were, visual demonstration accompanied by verbal instructions; guided movement; kinaesthetic perception and proprioception; tactile pressure, concurrent visual-motor feedback; visualisation, and mental practice to reinforce the movement pattern and a constraint led approach to prevent the mirror movements.[51].

The leg-yield ridden from the centre line towards the wall is a variation as well as a natural outcome of the head-to-wall leg-yield. This movement was not taught, but was added as a data point.

19. Blignault, K. Four Steps to Riding Success; J.A. Allen: London, UK, 2014.
51. Blignault, K.; Buys, T.; van Vollenhoven, E.; Ramkilawon, G. The Efficacy of the Four-Step Riding Programme to Facilitate Learning the Body Language Needed for Rider–Horse Communication. Master's Dissertation, University of Pretoria, Pretoria, South Africa, 2024.
58. Racinet, J.-C. Quit pushinng onto the bit. In Another Horsemanship–A Manual of Riding in the French Classical Tradition, 1994 ed.; Xenophon Press: Franktown, VA, USA, 1994; p. 1.
