# Supplementary material for: Teaching Direction Control in Horse Riding: A Pre–Post Intervention Proof-of-Concept Study Using Multi-Modal Motor Learning Techniques
Source: Animals (Basel). 2026 Oct 9;16(20):3170. doi: 10.3390/ani16203170 (PMC13700739; doi:10.3390/ani16203170)
Supplement: Supplementary file 1 [file animals-16-03170-s001.zip › Table S1 Horse-Rider Experience.pdf]

Horse–rider characteristics and reported problems

Name of Horse	Horse riding experience	Main problem	Rider experience	Comments
**Beta**	13-year-old Thoroughbred mare; raced for seven years and retired four years before the study.	Persistent racing anxiety; extremely forward-going, with little rhythm and balance.	21 years old; 12 years of riding experience with regular lessons from six riding teachers.	Hindquarters higher than the withers, which complicated engagement of the hindquarters.
**Delta**	7-year-old horse; retired from racing at three years of age, after which retraining commenced; owned by the current rider for four years.	Mild schooling problems, mainly distinct neck stiffness to the left and inconsistent flexion/contact.	28 years old; 20 years of riding experience with lessons from six riding teachers.	Least horse–rider communication problems of all participants; calm throughout the study; some pre-intervention ability to yield into a round flexion contact frame, but inconsistently.
**Digamma**	16-year-old Warmblood mare; previously owned and ridden for 10 years, mainly for hacking; ridden by the current rider for two years.	Extremely slow-moving with almost no response to forward leg cues; difficulty moving off light leg cues and developing a soft flexion frame of contact.	36 years old; 26 years of riding experience with lessons from three instructors.	Reduced response to leg cues was reported as apparently associated with continuous leg kicking by the previous owner.
**Epsilon**	9-year-old Clydesdale gelding – Thoroughbred cross gelding; ridden for five years.	Extreme resistance to being ridden in a round flexion frame of contact; limited lateral suppleness; not forward-going.	46 years of riding experience; 40 years of riding instruction from eight teachers.	
**Gamma**	4-year-old gelding; retired from racing seven months before the study and had undergone minimal retraining as a sport horse.	Anxious at the start of the study; none of the first three elements of the FEI training format were established pre-intervention.	21 years old; 14 years of regular riding experience with lessons from five riding teachers.	
