# Supplementary material for: Teaching Direction Control in Horse Riding: A Pre–Post Intervention Proof-of-Concept Study Using Multi-Modal Motor Learning Techniques
Source: Animals (Basel). 2026 Oct 9;16(20):3170. doi: 10.3390/ani16203170 (PMC13700739; doi:10.3390/ani16203170)
Supplement: Supplementary file 1 [file animals-16-03170-s001.zip › Table S2 MMLT Deviations.pdf]

MMLT technique	Deviation	Justification
**Mental practice**	Mental practice was used specifically for one participant	Mental practice is an established component of the FSRP but was not required by the other participants. This participant learnt primarily through physical movement; therefore, mental rehearsal of the required body and limb movements was used until she could reproduce the actual movement patterns required for the exercise
**Verbal instruction**	Verbal instruction was minimised for one participant and replaced mainly with visual demonstration and tactile, proprioceptive and kinaesthetic input	The participant experienced auditory processing difficulties, and verbal explanations did not adequately represent the physical movement patterns for her. Alternative sensory modalities were therefore emphasised to facilitate understanding and performance of the exercises
